# Summary of the best evidence for whole-course nutritional management of bladder cancer

**DOI:** 10.3389/fnut.2026.1916117

**Published:** 2026-08-13

**Authors:** Bingbing Zhang, Yuan Chen, Yan Wang, Yue Liu, Xiaohui Yu, Hongwen Xie

**Affiliations:** 1 Department of Nursing, The Fourth Affiliated Hospital of Jiangsu University, Zhenjiang, Jiangsu, China; 2 Department of Urology, Fuyang People’s Hospital, Fuyang, Anhui, China; 3 Department of Anesthesiology, Danyang People’s Hospital, Danyang, Jiangsu, China; 4 Department of Nursing, Zhenjiang Maternal and Child Health Hospital, Zhenjiang, Jiangsu, China

**Keywords:** bladder cancer, evidence summary, evidence-based, management, nutrition, whole-course

## Abstract

**Objectives:**

To synthesize the best evidence for whole-course nutritional management of bladder cancer (BC) and to obtain a structured summary of evidence for clinical guidance.

**Methods:**

A full search was conducted in multiple websites and databases (11 electronic databases, 10 guideline websites, and 14 major professional society websites) for expert consensus, expert recommendations, guidelines, evidence summaries, randomized controlled trials, and systematic reviews related to BC nutrition until December 2025, with a supplementary search in January 2026. The included studies underwent quality assessment, extraction and summary of evidence, and two-round Delphi expert consultation. A total of 17 experts were invited to participate in the first round, and 15 experts were invited in the second round.

**Results:**

Sixteen studies were included: one expert recommendation, one clinical decision, three expert consensus articles, four evidence summaries, and seven guidelines. We summarized 91 evidence statements across seven dimensions: nutrition team formation and management processes, three-tier nutritional diagnosis, principles of nutrition support therapy, content of nutritional interventions, nutritional surveillance, health education, and nutritional follow-up. The expert authority coefficient (Cr) was 0.88 for both rounds of consultation, and Kendall’s coefficients of concordance were 0.250 and 0.313, respectively, indicating acceptable expert authority.

**Conclusion:**

This study summarized the best evidence for whole-course nutritional management of BC across treatment methods and nutritional statuses. The findings lay an evidence-based basis for clinical practice, especially for patients with nutritional risk undergoing radical cystectomy.

**Systematic review registration:**

https://nursing.fudan.edu.cn/38724/list.htm, identifier ES20258783.

## 1 Introduction

Bladder cancer (BC) is the 10*^th^* most common malignancy globally, with about 614,000 new cases worldwide in 2022. It ranks 13*^th^* in mortality (1, 2), and the number of new deaths has been rising annually, with an increase up to 151.2% (3). Based on the tumor invasion depth into the bladder wall, BC includes muscle invasive and non-muscle invasive BC (4). Due to prolonged hematuria, BC patients often experience hypoalbuminemia, anemia, and malnutrition. Moreover, protein breakdown caused by surgery and discomfort resulting from chemotherapy (e.g., nausea, vomiting, diarrhea) all raise the nutritional risk. In particular, the postoperative incidence rates of gastrointestinal complications and intestinal obstruction are 20% and 14%, respectively, in patients undergoing radical cystectomy (RC), which further exacerbates the nutritional risk (5, 6). Research suggests that the incidence of malnutrition in BC is 16%–55%, and patients with preoperative malnutrition have an approximately 22% higher rate of postoperative complications, with an infection rate of 15% (7). Collectively, impaired nutritional status is a serious threat to the quality of life of BC patients. Nutritional management can improve patients’ nutritional status and facilitate postoperative rehabilitation (8, 9). However, only 33.7% of cancer patients with malnutrition are given nutritional interventions (10).

Nowadays, nutritional management of BC is conducted primarily in specific stages within the hospital, and a whole-course nutritional management bridging hospital, community, and home settings remain scarce. Consequently, it is difficult to monitor the nutritional status in real time post-discharge and promptly adjust follow-up measures, creating a management gap (11). Besides, the study population is limited, i.e., studies focus solely on patients undergoing RC, without covering radiotherapy, chemotherapy, and palliative care (12). In clinical practice, patients and their families lack sufficient knowledge regarding BC nutrition and hold some misconceptions, further compromising the efficacy of nutritional interventions. It is obvious that BC patients are faced with serious nutritional issues, and scientific and systematic nutritional management protocols are lacking.

Maximizing the efficacy of interventions via whole-course nutritional management is recommended by guidelinecs (13). Whole-course nutritional management is a systematic, dynamic, and individualized precision management pattern, where interventions are adjusted promptly based on the dynamic changes in nutritional status during hospitalization and post-discharge, continuously improving nutrition (14). Available studies have applied the whole-course nutritional management to patients undergoing surgery for urological tumors, and the results confirmed its effects of ameliorating postoperative nutritional status and outcomes, and facilitating recovery (15). Due to the prolonged course and complexity of BC, patients at different stages and undergoing different treatments have varying nutritional needs. Therefore, individualized precision nutritional interventions should be adopted based on disease progression, nutritional status, and treatment methods, necessitating a scientific, systematic, and precision nutritional management protocol.

Currently, relevant domestic and international guidelines and expert consensus focus primarily on treatment and medication and less on nutritional management (16, 17). Clinical medical workers’ capability in nutritional management and treatment remains insufficient. Therefore, scientific guidance for nutritional management and prognosis improvement is urgently needed. Using an evidence-based approach, this study intends to summarize the evidence for nutritional management at different stages of treatment for BC from high-quality domestic and international studies. The findings can provide medical workers with scientific and reliable evidence and references for the selection and formulation of interventions in clinical practice.

Unlike previous research focusing primarily on perioperative nutritional management in BC patients, we adopted a whole-course perspective covering from admission to post-discharge care, including surgery, radiotherapy, chemotherapy, and palliative care. Stratified management was implemented based on nutritional status, and patients were classified for the first time into four strata according to both treatment modality and nutritional status and received precise and individualized nutrition support. Additionally, we incorporated traditional Chinese medicine (TCM) techniques as adjunctive interventions to facilitate gastrointestinal functional recovery.

## 2 Materials and methods

### 2.1 Clinical question

We defined the clinical question by the PIPOST framework. P (Population): BC patients who undergo RC, radiotherapy, transurethral resection of the bladder, chemotherapy, or palliative care; I (Intervention): dietary guidance, nutrition education, enteral and parenteral nutrition, oral nutritional supplements (ONS); P (Professional): clinicians, dietitians, rehabilitation physicians, clinical pharmacists, and specialized nurses; O (Outcome): incidence of malnutrition; S (Setting): urology clinics, inpatient wards, and home. T (Study type): expert consensus, expert recommendations, guidelines, evidence summaries, systematic reviews, and randomized controlled trials.

### 2.2 Eligibility criteria

Inclusion criteria: (1) studies on the nutritional status of BC patients; (2) expert consensus, expert recommendations, guidelines, evidence summaries, randomized controlled trials, and systematic reviews; (3) formal or evidence-based consensus methods [guidelines with no strength of recommendation or evidence scoring were defined as having “no evidence base” (18)]; (4) the up-to-date version; and (5) Chinese- or English-language. Exclusion criteria: (1) duplicates, direct translations, or interpretations of existing guidelines; (2) unavailable full text; and (3) literature with quality assessment grades of C and D.

### 2.3 Search strategy

We performed a full search using subject headings plus free-text terms. English terms included but not limited to “Bladder Cancer,” “Urinary Bladder Neoplasms,” “Transurethral Resection of the Bladder,” “Radical Cystectomy,” “Nutrition,” and “Nutrition Management.” Corresponding Chinese terms were utilized in Chinese databases.

An information specialist from the university library with expertise in search methodology for systematic review assisted in developing the search strategy. Two reviewers (Xie Hongwen and Zhang Bingbing) who had received formal training in systematic review methodology drafted the initial strategy. To optimize sensitivity and specificity, the draft was subsequently refined by iterative pre-search and discussion with the research team and information specialist. The robustness of the strategy was guaranteed by (1) multiple rounds of pre-searches, (2) consultation with the information specialist, and (3) review and approval by the research supervisor (Xie Hongwen).

Using the “5S” pyramid of evidence-based resources, we performed a full search in multiple websites and databases, including one computerized decision-making system (UpToDate), 11 electronic databases (Embase, BMJ, PubMed, CINAHL, Web of Science, CNKI, Scopus, Cochrane, Sinomed, Wanfang, and JBI), 10 guideline websites (Registered Nurses’ Association of Ontario RNAO, Scottish Intercollegiate Guidelines Network SIGN, National Institute for Health and Care Excellence NICE, World Health Organization WHO, Agency for Healthcare Research and Quality AHRQ, New Zealand Guidelines Group NZGG, Guidelines International Network GIN, Yimaitong, National Comprehensive Cancer Network NCCN, and Australian Research Council NHMRC), and 14 major professional society websites (World Council of Enterostomal Therapists WCET, International Society of Urology SIU, European Associations of Urology EAU, European Society for Clinical Nutrition and Metabolism ESPEN, United Ostomy Associations of America UOAA, Wound, Ostomy and Continence Society WOCN, Canadian Urological Association CUA, Canadian Association for Enterostomal Therapy CAET, American College of Physicians ACP, American Urological Association AUA, American Society of Colon and Rectal Surgeons ASCRS, Chinese Urological Association, Chinese Urologic Surgeon Association CUSA, and the Cancer Nutrition Management Branch of the Chinese Society of Nutrition).

The search strategy is detailed in Supplementary Appendix A.

The search was conducted until December 2025, and an updated search was performed in January 2026 to identify any new publications, with one additional guideline included.

### 2.4 Screening and data extraction

Two reviewers independently conducted screening and data extraction. They were both trained in the JBI evidence-based methodology and experienced in systematic reviews. Disagreements were settled by consensus or consultation with a third reviewer when necessary.

After duplicate publications were removed by EndNote 20, the title and abstract were read based on the predefined eligibility criteria, and the full text was then examined. The studies excluded and the main reasons for exclusion are listed in Supplementary Appendix B. Besides, we checked the reference lists of all included studies to avoid any omissions. Using a standardized form, we extracted key data (title, year of publication, author/publisher, publication source, journal, and country/region).

### 2.5 Quality assessment

Two reviewers (Zhang Bingbing and Chen Yuan) independently scanned the titles and abstracts of all records retrieved, and the full texts of potentially relevant studies were obtained and assessed by the two reviewers. Disagreements were settled by discussion with a senior reviewer (Xie Hongwen). When conflicting evidence emerged, high-quality, evidence-based, and recently published studies were prioritized.

Clinical guidelines were assessed by the AGREE II covering 23 items in six domains: participant, rigor of development, scope and purpose, clarity of presentation, applicability, and editorial independence. Each item was scored on a seven-point scale (1: a lack of data or extremely poor quality, 7: a high-quality report meeting all criteria). The sum of the scores for all items represented the score for each domain. The raw score was standardized by the standard formula of AGREE II: standardized score (%) = (raw score–minimum possible score)/(maximum possible score–minimum possible score) × 100.

Since the criteria for standardized scores are not defined in the AGREE II User Manual, multiple quality assessment studies were extensively referenced (19–21). On this basis, the following recommendation criteria were defined: (1) Grade A (recommended): standardized scores ≥ 60% in all six domains; (2) Grade B (recommended with modifications): 30%–60% in most domains (≥3); (3) Grade C (not recommended): <30% in ≥3 domains. We included only guidelines of Grade A or Grade B. To ensure consistency, inter-rater reliability was quantified by the intraclass correlation coefficient (ICC): <0.40, poor; 0.40–0.75, moderate; >0.75, high (22).

Expert consensus and expert opinions were assessed by the JBI Critical Appraisal Checklist (23) from six items of credibility and logical development. A lower score corresponds to a higher level of evidence [Level 1 (the highest): evidence from systematic reviews of randomized controlled trials, Level 5 (the lowest): evidence from expert opinions or laboratory studies]. Two reviewers independently assessed each item as “Not Applicable,” “Yes,” “No,” or “Unclear.” Articles were included only if they met a predefined quality threshold, namely affirmative responses (“Yes”) to most items, emphasizing the logical derivation of conclusions. Evidence from UpToDate was directly considered high quality (24).

Four reviewers independently assessed the included guidelines and best practice guidelines, while two reviewers independently assessed the included expert consensus and recommendations.

### 2.6 Translation and summary of evidence

Recommendations regarding whole-course nutritional management of BC were acquired from the included studies by two reviewers. The results were subsequently cross-checked. Disagreements were settled by discussion or adjudication by a third reviewer. Both reviewers independently translated recommendations published in English into Chinese and compared them to reach a consensus. All recommendations (including both translations and original ones) were summarized according to the following principles: (1) for identical recommendations, the version with the clearest wording was selected (based on predefined criteria for completeness, accuracy, and clinical applicability); (2) for conflicting recommendations, their sources were traced, and the most recent and highest-quality evidence was prioritized; (3) for complementary recommendations, they were merged into a synthesized statement; and (4) for independent recommendations, the original statements were retained. This method was adapted to the established practice of evidence synthesis (25). During summary, genuine conflicts were rare. When conflicts emerged, they were typically settled by reviewing publication dates, the level of evidence, and consistency with broader public health principles.

### 2.7 Delphi expert consultation

Based on the best evidence for BC across treatment methods and nutritional statuses, a first draft of the whole-course nutritional management intervention protocols and an expert survey questionnaire were prepared. Inclusion criteria for experts: intermediate professional title or higher; Bachelor’s degree or higher; work experience of at least 10 years in urology, nutrition, or TCM, or less than 10 years but currently teaching at a university with a solid theoretical foundation; with specialized knowledge in relevant fields; with informed consent and active participation. Questionnaires were issued and collected via paper or email. A two-round consultation was conducted at an interval of 8 weeks, with a 1-week collection period for each round. The evidence was revised and refined based on expert opinions.

## 3 Results

### 3.1 Search results and overview

**FIGURE 1:**
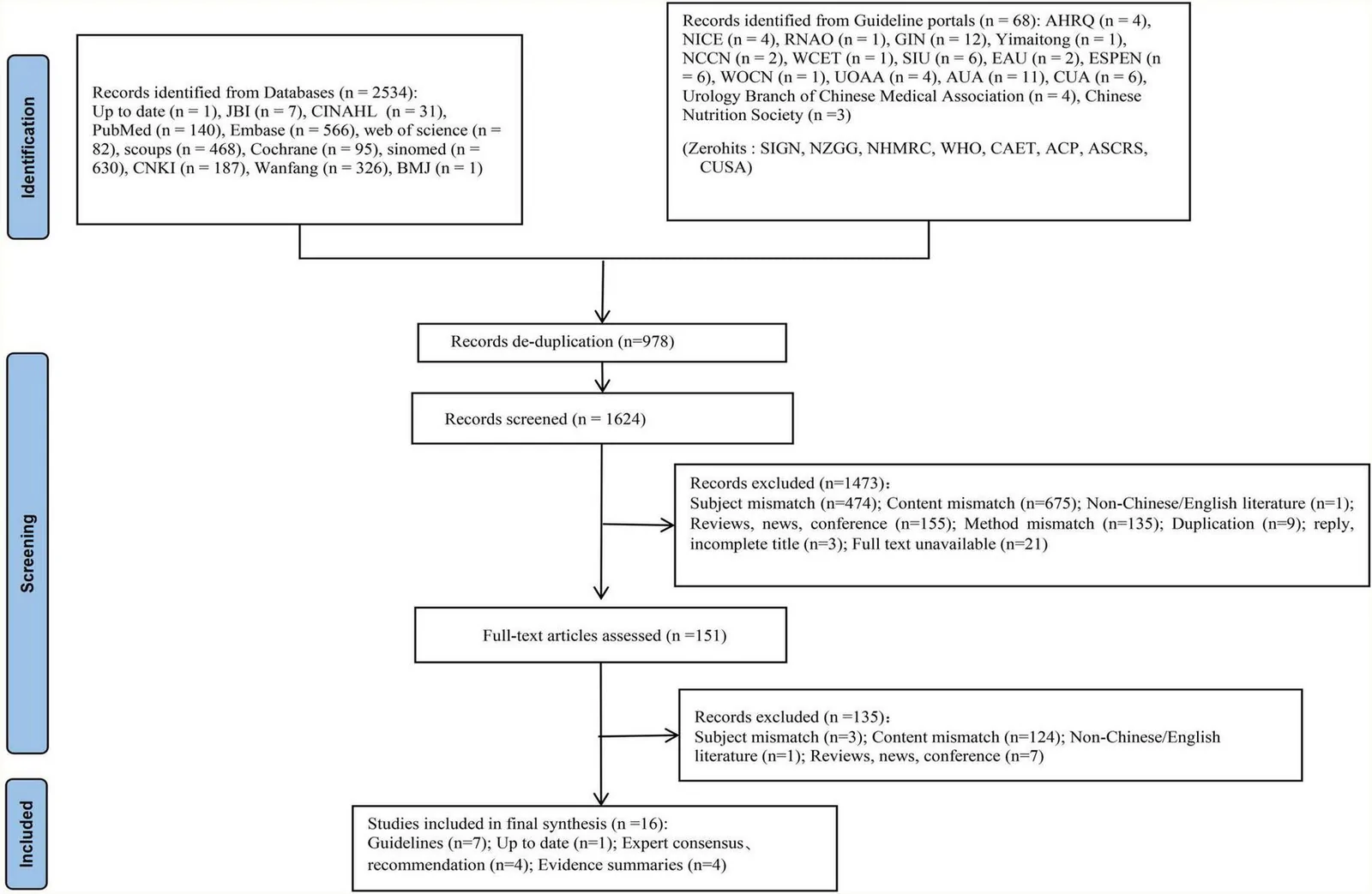
PRISMA flowchart.

We initially retrieved 2,602 records, of which 978 duplicates were removed. A total of 1,473 records were excluded following title and abstract reading, and another 135 were excluded after full-text review. A total of 16 studies were included finally, comprising one clinical decision, seven guidelines, three expert consensus articles, one expert recommendation, and four evidence summaries. The selection process is displayed in the PRISMA flowchart (Figure 1), and the basic study characteristics are listed in Table 1.

**TABLE 1:** Basic study characteristics (*n* = 16).

No	References	Year of publication/ update	Topic	Author/ publisher	Type	Country
1	Society of Parenteral and Enteral Nutrition, Chinese Medical Association (29)	2023	Clinical practice guidelines for enteral and parenteral nutrition in Chinese adult patients	CNKI	Guideline	China
2	Holzbeierlein et al. (16)	2024	Treatment of non-metastatic muscle-invasive bladder cancer	AUA	Guideline	U.S.
3	van der Heijden et al. (30)	2025	Guidelines on metastatic and muscle-invasive bladder cancer	EAU	Guideline	Europe
4	Muscaritoli et al. (28)	2021	Clinical nutrition in cancer	ESPEN	Guideline	Europe
5	Doris Grinspun et al. (43)	2019	Support for adults anticipating or living with an ostomy	RNAO	Guideline	Canada
6	The Academy of Nutrition and Dietetics (44)	2017	Oncology evidence-based guideline for nutrition practice in adults	PubMed	Guideline	U.S.
7	Lyu et al. (27)	2026	Clinical guidelines for nutritional therapy in cancer patients	CNKI	Guideline	China
8	Expert Collaborative Group on Enhanced Recovery After Surgery of the Chinese Bladder Cancer Consortium, Urology Branch of the Chinese Medical Association (45)	2018	Expert consensus on ERAS for radical cystectomy and urinary diversion	Wanfang	Expert consensus	China
9	Urological Health Promotion Branch, China Alliance for Healthcare Promotion (46)	2021	Consensus on safety of radical cystectomy + urinary diversion	CNKI	Expert consensus	China
10	Kang et al. (47)	2024	Expert consensus on the selection of ONS for cancer patients	Chinese Society of Nutrition	Expert consensus	China
11	Albisinni et al. (17)	2025	ERAS for patients undergoing radical cystectomy: Surgeons’ perspectives and recommendations 10 years after its implementation. European Journal of Surgical Oncology	European Journal of Surgical Oncology	Views and recommendations	Europe
12	Shariat et al. (48)	2025	Urinary diversion and reconstruction following cystectomy	UpToDate	Clinical decision	Austria
13	Xu et al. (49)	2025	Best evidence for rehabilitation management of urinary incontinence in bladder cancer after orthotopic neobladder reconstruction	PubMed	Evidence summary	China
14	Zhang et al. (12)	2023	Evidence summary on perioperative nutritional management of BC patients	Wanfang	Evidence summary	China
15	Long et al. (34)	2025	Evidence-based practice and future development of ERAS in urology: a GRADE-based multidimensional assessment	PubMed	Evidence summary	China
16	Dai et al. (41)	2025	Best evidence summary on gastrointestinal function recovery following radical cystectomy	PubMed	Evidence summary	China

### 3.2 Results of quality assessment

Four guidelines were assessed as Grade A and three as Grade B. The inter-rater reliability was high, with an ICC of 0.905–0.979 (>0.75) for all guidelines. The intermediate professional title or higher; Bechelor’s in Table 2.

**TABLE 2:** Results of quality assessment of included guidelines (*n* = 7).

No.	Percentage of standardized scores (%)						Number of domains ≥ 60%	Number of domains ≥ 30%	ICC	Level
	Scope and purpose	Participant	Rigor of development	Clarity of presentation	Applicability	Editorial independence				
(29)	100%	72.22%	96.35%	100%	80.20%	100%	6	6	0.974	A
(16)	100%	90.27%	98.96%	100%	84.38%	100%	6	6	0.907	A
(30)	100%	94.44%	93.23%	100%	81.25%	100%	6	6	0.979	A
(28)	100%	100.00%	95.31%	100%	74.00%	52.08%	5	6	0.968	B
(43)	100%	98.61%	100%	100%	87.55	100%	6	6	0.908	A
(44)	98.61%	61.11%	93.23%	90.28%	55.21%	72.92%	5	6	0.905	B
(27)	100%	76.38%	47.39%	100%	68.75	35.42%	4	6	0.979	B

### 3.3 Results of assessment of best practices, expert consensus, and recommended practices

All three expert consensus articles and one recommendation met the predefined quality threshold and were therefore incorporated to extract evidence. The results of quality assessment are detailed in Table 3.

**TABLE 3:** Results of quality assessment of expert consensus (*n* = 4).

Expert consensus/item	Item 1	Item 2	Item 3	Item 4	Item 5	Item 6	Overall assessment
	1	2	3	4	5	6	
(45)	Yes	Yes	Yes	Yes	Yes	Yes	Included
(46)	Yes	Yes	Yes	Yes	Yes	No	Included
(47)	Yes	Yes	Yes	Yes	Yes	Yes	Included
(17)	Yes	Yes	Yes	Yes	Yes	Yes	Included

^1^Item 1: Is the source of the viewpoint clearly indicated?

^2^Item 2: Is the viewpoint from influential experts in the field?

^3^Item 3: Does the proposed viewpoint focus on the interests of the relevant population?

^4^Item 4: Is the stated conclusion based on the analysis results? Is the expression of the viewpoint logical?

^5^Item 5: Are other existing references consulted?

^6^Item 6: Do the proposed viewpoint and previous literature have inconsistencies?

### 3.4 Summary of best evidence

We summarized the evidence for whole-course nutritional management of BC across seven key dimensions: nutrition team formation and management processes, three-tier nutritional diagnosis, principles of nutrition support therapy, content of nutritional interventions, nutritional surveillance, health education, and nutritional follow-up. This generated 91 distinct statements of best practice evidence, and they were scored by the JBI Evidence Pre-grading System (2014) (26) (section “2 Materials and methods). The evidence for nutritional management is displayed in Table 4.

**TABLE 4:** Best evidence for whole-course nutritional management of bladder cancer (BC).

Category	Evidence content			Numbers	Evidence level (grade)	Recommended Level
Nutrition team formation and	1. Establishment of the nutrition team	A multidisciplinary team consisting of clinical physicians, dieticians, rehabilitation physicians, clinical pharmacists, and specialized nurses shall be established to ensure the scientific validity of nutritional management (29).		1	5	B
nutritional management process		Patient family members or caregivers shall be incorporated to fulfill the objectives of patient monitoring and support (12).		2	5c	B
	2. Whole-course nutritional management process	It consists of five sequential steps: screening, assessment, diagnosis, intervention, and monitoring (29).		3	5	A
Three-tier nutritional diagnosis	1. Nutritional screening	1.1 Screening timing (28): 1.1.1 Nutritional risk screening shall be performed promptly upon the confirmed diagnosis of cancer patients. 1.1.2 For hospitalized patients, screening shall be initiated within 24 h after admission. 1.1.3 For those with no identified nutritional risk in the initial screening, a re-screening is recommended 1 week later.		4	5	A A B
		1.2 Screening tool: 1.2.1 Recommended nutritional risk screening 2002 (NRS 2002) (12).		5	3c	
		1.2.2 Modified nutrition risk in the critically ill (m-NUTRIC) tool for critically ill patients (29).		6	3	
	2. Nutritional assessment	2.1 Target population for assessment: nutritional assessment shall be conducted for cancer patients with confirmed nutritional risk (44).		7	5	A A A B A
		2.2 2.2.1 Assessment components: including dietary assessment, body composition (e.g., subcutaneous fat loss and muscle wasting), and nutrition impact symptoms (NIS). Laboratory tests [including inflammatory and metabolic markers, e.g., hypoalbuminemia (<3.5 g/dL)] may be repeatedly assessed throughout the treatment course. 2.2.2 The patient’s tumor treatment status, comorbidities, cancer cachexia, and psychosocial and economic factors (e.g., social support) are assessed (28, 29, 44).		8	5	
		3 Assessment instruments: 3.1 Preferred assessment instrument: Patient-Generated Subjective Global Assessment (PG-SGA) (27).		9	5	
		3.2 Assessment instruments for critically ill patients: acute gastrointestinal injury (agi) assessment scale and agi ultrasound (agius) scoring system (29).		10	5	
		3.3 Global leadership initiative on malnutrition (GLIM) (29). 3.4 For patients at nutritional risk, nutritional assessment should be performed based on three phenotypic indicators [involuntary weight loss, low body mass index (BMI), and reduced muscle mass] and two etiologic indicators (reduced food intake or malabsorption, disease or inflammation) (27).		11	5	
	3. Comprehensive evaluation	3.1 Types of malnutrition and nutritional treatment regimens should be analyzed from four dimensions: energy expenditure level, stress severity, inflammatory burden, and metabolic status (27): 3.1.1 Energy expenditure is primarily determined by six factors: age, sex, body weight, tumor burden, physical activity level, and treatment status. 3.1.2 Indicators reflecting the body’s stress severity: insulin resistance index, C-reactive protein (CRP), triglycerides, and C-reactive protein, triglyceride, glucose index (CTI). 3.1.3 The body’s inflammatory status can be evaluated via cytokine responses (TNF-α, IL-1) or the inflammatory burden index (IBI). 3.1.4 Indicators reflecting metabolic status include low-density lipoprotein cholesterol (45.7%), total cholesterol (34.2%), and prealbumin (30.0%).		12	5	A
Principles of nutritional support therapy	There are three items in total, whose practical implementations are non-conflicting and mutually complementary. 1. Principle 1: stratified by the patient’s nutritional status: 2. 1.1 For patients with no malnutrition or mild malnutrition: anti-tumor therapy shall be administered concurrently with nutritional education; 3. 1.2 For patients with moderate malnutrition: anti-tumor therapy shall be initiated in parallel with medical nutrition therapy (MNT). 4. 1.3 For patients with severe malnutrition: medical nutrition therapy shall be prioritized for 1–2 weeks, followed by the initiation of anti-tumor therapy while continuing MNT. Regardless of the presence or absence of malnutrition, all patients shall undergo re-nutritional diagnosis after completing one course of anti-tumor therapy (27).			13	5	A
	5. Principle 2: guided by the intensity of nutritional support: The first-line option for nutritional support shall be dietary counseling plus nutritional education, followed by stepwise escalation to diet plus oral nutritional supplements (ONS), total enteral nutrition (TEN), partial parenteral nutrition (PPN), and total parenteral nutrition (TPN). These modalities may be utilized either alone or in combination, depending on the patient’s clinical status (27, 29).			14	5	A
	6. Principle 3: nutritional transition guided by energy requirements (27): 3.1 For patients with oral intake capability, the first tier shall be prioritized. The upward transition follows the 60% principle: 1) When the current tier fails to meet 60% of the target nutritional requirements, the next higher tier shall be adopted (e.g.; 2) If nutritional education cannot meet 60% of the target nutritional requirements, ONS shall be initiated; 3) If ONS is insufficient, TEN shall be selected; 4) If TEN fails to meet 60% of the target nutritional requirements, enteral nutrition plus PPN shall be used; 5) If enteral nutrition plus PPN still cannot meet 60% of the target nutritional requirements, total TPN shall be chosen. 3.2 For patients with complete loss of oral intake capability, the fifth tier shall be initiated first, followed by downward transition in accordance with the 50% principle: 1) When the next lower tier can meet 50% of the target nutritional requirements, the current tier shall be gradually degraded, while the next lower tier shall be progressively escalated (e.g., if enteral nutrition can meet 50% of the target nutritional requirements, parenteral nutrition shall be gradually reduced with a concurrent progressive increase in enteral nutrition. If oral nutrition can meet 50% of the target nutritional requirements, tube feeding shall be gradually reduced, while oral intake shall be progressively increased. If regular diet can meet 50% of the target nutritional requirements, medical nutrition shall be gradually reduced with a concurrent progressive increase in regular dietary intake); 2) The recommended observation period for nutritional transition is 3–5 days for general patients and 2–3 days for critically ill patients. 3.3 The recommended observation period for nutritional transition is 3–5 days for general patients and 2–3 days for critically ill patients.			15	5	A
Specific content of nutritional intervention						
(⇁)Nutritional Intervention for bladder cancer patients without nutritional risk: predominantly focused on dietary counseling and nutrition education						
Patients not undergoing RC	1. Dietary principles: prioritize a dietary pattern dominated by vegetables, fruits, and whole grains, with reduced intake of saturated fats, red meat, and alcohol (28).			16	5	A
	2. Dietary guidelines 2.1 It is recommended that the daily diet adhere to the 16-character principle: “Diverse Selection, Controlled Total Intake, Improved Nutritional Quality, and Timing Mastery.” Specifically, individuals should consume at least 20 food varieties daily and no fewer than 30 varieties weekly. The daily intake of vegetables and fruits should be at least 5 servings (2.5–3 servings of vegetables and 1.5–2 servings of fruits). Additionally, overly salty foods and sugar-sweetened beverages should be avoided, while the intake of anti-inflammatory foods (e.g., deep-sea fish) should be increased (16).			17	5	A
	2.2 It is recommended that a personalized meal plan be formulated for patients under the supervision of a clinical dietician, with the implementation of small, frequent meals at regular intervals and fixed portions. Additionally, patients should be encouraged to consume food in a pleasant environment to enhance dietary enjoyment and treatment adherence (27).			18	5	A
	2.3 It is recommended that energy intake align with that of healthy participants, ranging from 25 to 30 kcal/(kg⋅d). For patients with normal liver and renal function, protein intake should be 1–1.5 g/(kg⋅d); for those with concurrent critical illness, 1.5–2.0 g/(kg⋅d) may be administered based on clinical requirements (29). The provision of vitamins and minerals should approximate the daily recommended intake (DRI), and the use of high-dose micronutrients is not advised (28, 47).			19	5	A
	3. It is recommended that individuals maintain a healthy weight (BMI 18.5–25 kg/m^2^) and adopt a healthy lifestyle, engage in regular physical activity, participate in aerobic exercise, and incorporate individualized resistance training to preserve muscle strength and muscle mass (28).			20	5	A
**Patients undergoing RC**	**1. Preoperative nutritional intervention**	1.1 It is recommended to adopt an enhanced recovery after surgery (ERAS) approach during the perioperative period, including preoperative carbohydrate loading, goal-directed fluid therapy, and multimodal analgesia (17, 28, 34).		21	5	A A A B B
		1.2 Quit smoking and drinking 4 weeks before surgery (17).		22	5	
		1.3 **Recommendation against routine mechanical bowel preparation preoperatively** (34).		23	2	
		1.4 Avoiding intestinal antibiotics, laxatives such as mannitol or polyethylene glycol electrolyte solutions can be administered the day before surgery. However, for patients with severe constipation, thorough mechanical bowel preparation combined with oral antibiotics is recommended prior to surgery (45).		24	4	
		1.5 Overnight fasting is not recommended (29). Soft food may be consumed up to 6 h before surgery, and clear liquids (transparent fluids) up to 2 h before (17, 34).		25	5	
	**2. Postoperative nutritional intervention**	2.1 Routine placement of a nasogastric tube before or after surgery is not recommended (45); if placement is necessary, it should be removed within 4 h after surgery (34).		26	5	A A B A A A A
		2.2 Within 24 h postoperatively (the early postoperative period), oral intake of nutritional liquids may be initiated once intestinal flatus is passed or bowel sounds are restored (12, 29).		27	5b	
		2.3 For patients receiving oral intake, reduce the amount of prescribed diet when nausea occurs. If nausea persists, decrease food intake; if vomiting occurs, suspend feeding for 8 h and then resume with a liquid diet. If vomiting continues, gastrointestinal decompression is required, and standard diet should be resumed only after tube removal (12).		28	1b	
		2.4 A multimodal prophylactic regimen combining a 5-hydroxytryptamine type 3(5-HT_3_) receptor antagonist with dexamethasone (4 or 8 mg) is recommended for the prevention of nausea and vomiting (34).		29	5	
		2.5 Pain management: a multimodal non-opioid analgesic strategy is recommended, including acetaminophen and/or ketorolac (only as a breakthrough pain medication) (30).		30	5	
		2.6 Thromboprophylaxis: it is recommended to provide pharmacological thromboembolic prophylaxis during the perioperative period, starting on the first postoperative day, with at least 4 weeks of continuous venous thromboembolism prophylaxis using medications such as low-molecular-weight heparin (30).		31	3	
		2.7 Following postoperative regain of consciousness, patients may assume a semi-recumbent position or perform moderate in-bed activities—a 6-h pillow-free flat supine position is not required. Standing or chair-sitting is encouraged within 24 h postoperatively; ambulation should be initiated on the second postoperative day. Daily activity targets should be established, with gradual escalation of activity volume (41).		32	1	
		2.8 Mucus secretion from the neobladder typically peaks at approximately 7 days postoperatively. To prevent neobladder mucus obstruction, regular monitoring of urinary drainage patency is recommended, and irrigation with sodium bicarbonate solution should be performed as a prophylactic measure (46).		33	5	A
(⇁)Nutritional intervention for bladder cancer patients at nutritional risk: strengthening nutritional support therapy based on bladder cancer-specific dietary counseling and nutrition education						
Patients not undergoing RC	1. Individualized nutritional support	. 11. Develop and implement an individualized nutritional support therapy plan based on the nutritional status, gastrointestinal function, and disease severity of cancer patients (47); nutritional support should ideally be initiated before the onset of severe malnutrition in patients (28).		34	5	A A B A A B A
		1.2 For patients with malnutrition or at risk of malnutrition, unrestricted dietary energy intake is recommended (28).		35	5	
		1.3 For critically ill patients with high nutritional risk or severe malnutrition, initiating moderate feeding (50%–70% of the target volume) at the onset of nutritional therapy may yield clinical benefits; it is recommended to achieve 80% of the estimated target energy and protein intake within 48–72 h while preventing refeeding syndrome (RS) (29).		36	5	
		1.4 For weight-loss patients with insulin resistance, it is recommended to increase the fat-to-carbohydrate energy ratio to elevate energy density and reduce glycemic load (28). For patients with long-term malnutrition complicated by hypophosphatemia and hypokalemia, electrolyte disturbances should be corrected, and vitamin B_1_ (200–300 mg per day) plus balanced micronutrients should be supplemented; meanwhile, potassium (approximately 24 mmol/kg/d) and phosphate (approximately 0.3–0.6 mmol/kg/d) should be monitored (28).		37	5	
		1.5 For anemic patients with good tolerance, iron supplementation is recommended (30).		38	1	
		1.6 For patients undergoing chemotherapy who are at risk of weight loss or nutritional risk, supplementation with omega-3 polyunsaturated fatty acids (ω-3PUFA), long-chain n-3 fatty acids, or fish oil is recommended to stabilize or improve appetite, increase food intake, muscle mass, and body weight, and mitigate chemotherapy-induced adverse reactions (28, 29).		39	5	
		1.7 It is recommended that during radiotherapy, adequate nutritional intake be ensured via individualized nutritional counseling and/or the use of ONS. Parenteral nutrition (PN) should be administered in cases of nutritional inadequacy (e.g., severe radiation enteritis or profound malabsorption), yet PN is not recommended as a routine intervention for radiotherapy (28).		40	5	
	2. Nutritional support therapy—oral nutritional supplements (ONS)	2.1 Target population: for patients with inadequate food intake (e.g., intake < 50% of the required amount for 1 consecutive week, or intake ranging from 50% to 75% of the required amount for more than 2 weeks), ONS are recommended (28).		41	5	A A
		2.2 Energy requirements: for elderly bladder cancer patients at nutritional risk, it is recommended that they receive ONS providing a minimum of 400 kcal of energy and 30 g of protein daily, with close monitoring of ONS tolerance during administration (47).		42	5	
		2.3 Formulation selection: on the basis of the nutritional diagnosis and treatment process, individualized implementation and timely adjustments should be conducted in alignment with the clinical characteristics of cancer patients to reduce the incidence of complications (47): 2.3.1 In most cases: standard whole-protein formulas are recommended. 2.3.2 For patients with severe intestinal dysfunction: elemental formulas (amino acid- or short peptide-based) should be selected. 2.3.3 For patients with diabetes mellitus or impaired glucose tolerance: diabetes-specific formulas (with an increased fat-to-carbohydrate energy ratio to reduce glycemic load) are indicated. 2.3.4 For patients with chronic kidney disease (CKD) complicated by severe electrolyte abnormalities: formulas low in sodium, potassium, and phosphorus are preferred. 2.3.5 For patients requiring fluid restriction: high-energy-density formulas should be chosen to meet energy requirements while minimizing fluid load. 2.3.6 For patients with diarrhea or constipation: fiber-containing formulas are recommended; routine combination with probiotics is not advised.		43	5	A
	3. Key focuses of nutritional management for patients with advanced bladder cancer	3.1 Nutritional support for patients with advanced bladder cancer should be comprehensively determined based on prognosis, expected survival duration, and tumor activity (28): 3.1.1 For patients with an expected survival duration of weeks to months: non-invasive interventions should be adopted, with a focus on psychosocial support. 3.1.2 For patients with an expected survival duration of at least several months, favorable prognosis, low tumor activity, and C-reactive protein (CRP) < 10 mg/dL: Comprehensive nutritional counseling and support should be provided. 3.1.3 For patients with an expected survival duration of months to years: nutritional therapy should be administered to ensure adequate energy and protein intake and reduce metabolic disorders.		44	3	B B B
		3.2 It is recommended that progesterone, corticosteroids, and other agents be considered for appetite enhancement in advanced cancer patients with anorexia; however, attention should be paid to potential adverse effects, including muscle atrophy, insulin resistance, infections, and thromboembolism (28).		45	5	
		3.3 For patients with early satiety, it is recommended to administer prokinetic agents following the diagnosis and treatment of constipation; however, attention should be paid to the adverse effects of metoclopramide and domperidone (28).		46	5	
		3.4 For patients with cancer cachexia, priority should be given to monitoring nutrition-impacting symptoms, inflammatory markers [e.g., elevated C-reactive protein (CRP)], and fluid accumulation; effective measures should be implemented immediately to alleviate symptoms, prevent or slow the loss of body fat and body weight (44).		47	5	A A
		3.5 For terminally ill patients, priority should be given to comfort enhancement (e.g., oral care), with brief and limited fluid replacement and small-volume feeding; intravenous fluid therapy may be attempted to improve or maintain cognitive function (28).		48	5	
Patients undergoing RC	1. Preoperative nutritional intervention	1.1 A 2–6 weeks multimodal prehabilitation program implemented preoperatively (encompassing physical exercise, nutritional support, and psychological intervention) has been shown to enhance patients’ health status (29, 30).		49 50 51	1 3 1b	A B B
		1.2 In patients with severe malnutrition, nutritional support should be initiated 7–10 days preoperatively (45).				
		1.3 For patients, immune-nutrition supplements may be additionally administered 5–7 days preoperatively and 5–7 days postoperatively, at a frequency of 2–3 times per day. An immune-enhancing ONS formula should be selected, which contains one or more of the following components: glutamine, arginine, nucleotides, vitamin A, and omega-3 fatty acids (fish oil) (12, 47).				
		1.4 Preoperative optimization of nutrition-related conditions, including hypertension, diabetes mellitus, and anemia, is recommended (17), with target blood glucose levels maintained below 10 mmol/L (34).		52	5	A A
		1.5 Oral administration of 800 and 400 ml of 12.5% carbohydrate solution (such as glucose or maltodextrin) the night before surgery and 2–4 h prior to anesthesia induction, respectively, helps reduce catabolic response and improve postoperative insulin resistance (17, 29, 34).		53	5	
	**2.** Postoperative nutritional intervention	2.1 During the acute postoperative phase, patients should avoid an excessively high carbohydrate-to-fat ratio to prevent exacerbation of glucose metabolism disorders. Fat-derived energy typically accounts for 30%–50% of non-protein calories. The recommended daily intake is 1.0–1.5 g⋅kg^–1^ of fat and 3.0–5.0 g⋅kg^–1^ of glucose, with a carbohydrate-to-fat ratio generally maintained at 60/40 or 70/30; in critically ill patients, this ratio may be adjusted to 50/50 (29).		54	5	A A A A A A A A
		2.2 For patients requiring fasting, adequate intravenous nutritional support should be administered (46), while fluid overload must be avoided. Goal-directed fluid therapy is recommended, and balanced salt solutions containing synthetic colloids are a viable option (45).		55	5	
		2.3 Measures for promoting postoperative gastrointestinal recovery	2.3.1 Chewing gum: patients should chew gum at least three times daily starting from the first postoperative day until the recovery of intestinal peristalsis (41, 45).	56	1	
			2.3.2 Patients without contraindications may receive low-dose opioid antagonist therapy to accelerate gastrointestinal recovery (16).	57	5	
			2.3.3 At the conclusion of RC, regardless of the type of urinary diversion implemented, external drainage via ureteral stents is recommended to optimize upper urinary tract drainage and facilitate gastrointestinal recovery (17, 41).	58	1	
			2.3.4 For established intestinal obstruction, fasting and gastrointestinal decompression are indicated. Low-level intestinal obstruction may be managed via colonoscopic tube placement. In cases of intestinal obstruction refractory to conservative treatment, early abdominal exploration should be performed to diagnose and address the underlying etiology (46).	59	5	
		2.4 Nutritional support therapy—enteral nutrition (EN)	2.4.1 Target population: patients who are unable to eat orally independently and are expected to achieve < 60% of the target intake for more than 7 days may be administered EN within 24 h postoperatively; however, EN is contraindicated in patients with upper gastrointestinal bleeding, intractable vomiting, complete intestinal obstruction, or severe gastrointestinal motility dysfunction (29).	60	5	
			2.4.2 Formulation selection: ➀ It is recommended to use an EN formula containing dietary fiber and ω-3 polyunsaturated fatty acids (ω-3 PUFA), with fat contributing 50% of total energy. For patients with persistent diarrhea complicated by severe malabsorption or those unresponsive to dietary fiber, a short peptide-based formula is indicated (29). ➁ Home-prepared diets are not recommended (29). ➂ For diarrhea occurring during EN therapy, underlying disease or non-nutritional drug-related causes should be ruled out first, rather than discontinuing EN (29).	61	5	
			2.4.3 Route of infusion: ➀ Nasogastric tube is the first choice for enteral nutrition (EN) administration (28). During tube feeding, the head of the bed should be elevated at 30∼45°(29). ➁ It is recommended to administer EN via a continuous infusion pump, with the infusion rate adjusted based on the patient’s tolerance (e.g., nausea, vomiting, abdominal distension, diarrhea). However, 24 h continuous infusion should be avoided in patients at high risk of aspiration (29).	62	5	A A A
			2.4.4 Consideration: ➀ Initial oral nutritional intake should be adjusted based on the patient’s gastrointestinal function status and individual tolerance. For patients with impaired gastrointestinal function, close monitoring and assessment of their tolerance to EN are recommended (29).	63	5	
			➁ Elderly patients aged > 70 years and those with severe senile dementia should be promptly evaluated for the risk of aspiration (29).	64	3	
		2.5 Nutritional support therapy—parenteral nutrition (PN)	2.5.1 **Target population:** For patients with low nutritional risk (3 ≤ NRS 2002 < 5 or NUTRIC < 6), if EN support fails to reach 60% of the target feeding volume after 7 days of treatment; for patients with high nutritional risk (NRS 2002 ≥ 5 or NUTRIC ≥ 6), supplementary parenteral nutrition (SPN) is recommended if EN cannot meet 60% of the body’s energy and protein requirements within 48–72 h (27, 29); for severely malnourished patients with severe gastrointestinal dysfunction who are unable to receive EN, early initiation of total TPN is recommended (29).	65	5	A A A B A
			2.5.2 Formulation selection: ➀ It is recommended to use “all-in-one” formulations, namely total nutrient admixtures (TNA), where various nutritional components are fully mixed to achieve a rational ratio (27, 29). ➁ Fat emulsions, glutamine (Gln) dipeptides, and other components may be added to PN based on the patient’s metabolic status, immune function, gastrointestinal function, and infection status. However, Gln dipeptide supplementation is not recommended for critically ill patients with severe liver or renal dysfunction and shock (29). ➂ A standard dose of multivitamins (including essential fat-soluble and water-soluble vitamins), trace elements, and electrolytes should be incorporated into PN. During the addition process, the compatibility with other components in the nutrient solution and the stability of the formulation must be taken into consideration (29).	66	5	
			2.5.3 Route of infusion: ➀ Central venous vascular access devices are recommended as the primary infusion route for PN (29).	67	5	
			➁ The cyclic infusion method is recommended; when conditions permit, an infusion pump should be used to control the infusion rate (8–20 h/d), and a portable pump is recommended for patients receiving home parenteral nutrition (HPN) (29).	68	5	
			2.5.4 Considerations: ➀ For patients requiring strict restriction of fluid and electrolyte intake, with severe metabolic disturbances, or with specific nutritional and fluid volume requirements, it is recommended to administer hospital-prepared individualized PN prescriptions. ➁ Human albumin infusion may be administered when necessary to alleviate anastomotic edema (46).	69	5	
			➂ During nutritional therapy for critically ill patients, blood glucose fluctuations are pronounced; blood glucose monitoring and insulin therapy are essential interventions, and insulin dosage can be precisely regulated via a microinfusion pump (29). ➃ Standardized selection of catheter access routes and standardized maintenance practices are recommended to prevent PN-related infections, with early initiation of enteral feeding; prophylactic administration of antibiotics is not recommended, and hepatoprotective agents should be used rationally (29).	69	5	A
		2.6 Adjunctive intervention of traditional Chinese medicine (TCM)	2.6.1 External TCM therapies, including acupuncture, acupressure, auricular therapy, electroacupuncture, and music therapy, are recommended to alleviate preoperative anxiety and fear, as well as to promote postoperative gastrointestinal recovery. For instance, auricular compression and acupoint sticking may be applied 1 day preoperatively and 2–3 days postoperatively (41).	70	1	B B B
			2.6.2 Acupoint stimulation is recommended to be initiated from the first postoperative day, with each session lasting 30 min for a consecutive 3-day course. The target acupoints include bilateral Zusanli (ST36), Shangjuxu (ST37), Sanyinjiao (SP6), and Hegu (LI4), aiming to promote gastrointestinal recovery (41).	71	1	
			2.6.3 It is recommended that patients with postoperative ileus receive a combined intervention of TCM and modified oral traditional herbal formulas, such as Dachengqi Decoction, Xiaochengqi Decoction, Simo Decoction, and Tongfu Decoc (41).	72	4	
Nutritional monitoring	1. Bladder cancer patients with nutritional risk should undergo routine and regular nutritional monitoring before and after nutritional support therapy (29, 46).			73	5	A
	2. Staged dynamic monitoring of nutritional status (27): 2.1 It is recommended to monitor rapidly changing indicators weekly, including laboratory parameters such as complete blood count, electrolytes, liver function, kidney function, inflammatory markers (IL-1, IL-6, TNF, CRP), nutritional panels (albumin, prealbumin, transferrin, retinol-binding protein, free fatty acids), and blood lactate. 2.2 It is recommended to assess intermediate outcome measures—such as anthropometric parameters, body composition, quality of life, physical function (e.g., usual gait speed test, timed up and go test, stair climb test, and grip strength test), and tumor lesions—every 4–12 weeks. 2.3 It is recommended to assess the slow-changing indicator—survival time—once a year.			74	5	A
	3. A standardized NIS scale should be developed, and patients or caregivers are recommended to conduct self-assessment or proxy assessment, followed by recording changes in the number of NIS, once every 2 weeks (27).			75	5	A
	4. For surgical patients, regular monitoring of acid-base status is recommended, with special attention paid to hyperchloremic metabolic acidosis. For patients with a serum bicarbonate level < 21 mmol/L, oral sodium bicarbonate supplementation should be considered, and calcium and vitamin D supplementation should also be provided (48).			76	1c	A
	5. All patients undergoing urinary diversion with an ileal conduit, especially long-term survivors, should be considered for regular monitoring of vitamin B12 levels (48).			77	3c	A
Health education	1. Healthcare professionals should provide personalized nutrition education to patients through diverse formats (oral communication and written materials), including ostomy-related education (17, 34).			78	3	A
	2. Preoperative nutritional education ≥ 3 times per month (27).			79	4b	A
	3. Patients and caregivers learn simple yet effective techniques for evaluating the outcomes of nutritional therapy to conduct self-assessment of treatment effectiveness (27).			80	5	A
	4. Lifestyle modifications should be implemented, including ensuring adequate sleep, avoiding overexertion, and establishing habits of oral nutritional supplementation and physical activity (27).			81	5	A
	5. For overweight and obese individuals (BMI > 30 kg/m^2^), a 5% body weight loss should be achieved and maintained, with a reduction in caffeine intake (49).			82	1	A
	6. Weight monitoring should be conducted once every 1–2 weeks, with measurements taken in the morning upon waking, after voiding the bladder and bowels, and while wearing light clothing; the results should be recorded. Any unexplained weight loss exceeding 2% warrants consultation with the attending physician or an outpatient visit (27).			83	5	A
	7. A daily fluid intake of approximately 2,000 mL should be maintained, and a high-protein, high-carbohydrate diet should be adopted to prevent constipation (12).			84	3c	B
	8. For patients with insomnia, fluid intake should be restricted within 3 h prior to bedtime (49).			85	5	B
	9. For patients with chronic insufficient dietary intake or malabsorption, home EN or PN is recommended for eligible individuals. Prior to the initiation of a home PN training program, it is imperative to assess the patient’s cognitive and physical capabilities (28).			86	5	A
	10. For surgical patients, preoperative stoma site marking should be performed, and preoperative guidance on abdominal pressure training and pelvic floor muscle rehabilitation training should be provided (27). Lifting of objects exceeding 10 pounds (1 pound = 0.454 kg) should be avoided within 1 month postoperatively, while abdominal exercises, pelvic tilts, and knee rotations may be initiated 3 months after surgery (43).			87	5	A
	11. Patients should be instructed to use a voiding diary to document their urination patterns over 3–7 days, with objective measurements of average voided volume, daytime and night-time urination frequency, and the presence of urinary incontinence episodes (49).			88	2	A
Nutritional follow-up	1. All patients should undergo nutritional assessment prior to discharge. For patients at risk of malnutrition or with established malnutrition, continuation of appropriate nutritional support is recommended post-discharge; ONS are the preferred option, administered on the basis of dietary guidance, with the duration determined by the patient’s nutritional status and subsequent treatment trajectory (28, 29).			89	5	A
	2. All surgical patients should undergo telephone follow-up within 24–48 h post-discharge to assess recovery status and establish a rapid readmission pathway (41).			90	5	A
	3. All patients with severe malnutrition should undergo regular hospital follow-up visits or telephone-based nutritional follow-up post-discharge, with a frequency of at least once every 3 months (27).			91	5	A

### 3.5 Delphi expert consultation

(1) Analysis of general information of experts: Experts’ academic backgrounds and professional titles were described by proportions and frequencies. Quantitative data were described by mean ± standard deviation. Two-round expert consultation was conducted, involving 17 experts in the first round, and 15 experts in the second round. General information of experts is shown in Table 5.

**TABLE 5:** Experts’ general information.

Item	Category	Round 1 (*n* = 17)		Round 2 (*n* = 15)	
		Number	Percentage (%)	Number	Percentage (%)
Sex	Male	10	58.82	9	60
	Female	7	41.18	6	40
Age	30–40	4	23.53	2	13.33
	40–50	9	52.94	9	60
	>50	4	23.53	4	26.67
Professional title	Intermediate	4	2.53	2	13.33
	Associate senior	9	52.94	9	60
	Full senior	4	23.53	4	26.67
Years of working	<10	1	5.88	0	0
	10–20	4	23.53	2	13.33
	20–30	8	47.06	8	53.33
	>30	5	29.41	5	33.33
Work	Urology physician	6	35.29	5	33.33
	Urology nurses	4	23.53	3	20
	Nurses	1	5.88	1	6.67
	Nutrition physician	1	5.88	1	6.67
	Obstetrics and gynecology	1	5.88	1	6.67
	Emergency and critical care	1	5.88	1	6.67
	Cardiology	1	5.88	1	6.67
	TCM	1	5.88	1	6.67
	Orthopedic sports	1	5.88	1	6.67

(2) In the first round of the survey, thirty questionnaires were issued, of which 17 were collected and confirmed as valid, with a valid response rate of 57%. A total of 13 experts provided feedback. In the second round, 17 questionnaires were issued, of which 15 were collected, with a valid response rate of 88%. Three experts provided feedback. Expert authority was measured by the authority coefficient (Cr), which was calculated by the coefficient of expert familiarity (Cs) and the coefficient of judgment basis (Ca): Cr = (Ca + Cs)/2, with Cr ≥ 0.7 considered acceptable. The Cr was 0.88 (>0.7), suggesting high expert authority and reliable consultation conclusions. The Kendall’s coefficients of concordance were 0.250 and 0.313, respectively, for the two rounds, indicating that the concordance in the second round improved over the first. The expert opinions were summarized, similar items were consolidated, and duplicates were removed. The research team discussed and responded to the expert opinions, then provided anonymous feedback to all experts for further consultation until opinions had no significant changes. The Ca, Cr, Cs, and summary and revision of expert opinions are tabulated in Supplementary Appendix C. The importance scores and feasibility scores for the 91 evidence statements are presented in Supplementary Appendix D.

## 4 Discussion

### 4.1 Overall logic of the evidence summary framework

The primary indicators of this summary (nutrition team formation and management processes, three-tier nutritional diagnosis, principles of nutrition support therapy, content of nutritional interventions, nutritional surveillance, health education, and nutritional follow-up) followed the logic of “organizational support → process guideline → diagnostic grading → treatment principle → intervention implementation →surveillance and follow-up.” The first round of consultation received endorsement from 17 experts, with a Cr of 0.88. The Kendall’s coefficients of concordance were 0.250 and 0.313, respectively, for the two rounds (*P* < 0.01), indicating statistically significant but low-to-moderate coordination and acceptable content validity.

The secondary indicators were obtained within the primary indicator framework combined with disease stage and nutritional risk. The three-tier nutritional diagnosis system comprised screening, assessment, and comprehensive evaluation (27). Nutritional Risk Screening 2002 (NRS2002) (for general patients) and modified Nutrition Risk in the Critically Ill (m-NUTRIC) (for critically ill patients) were recommended for screening. Patient-Generated Subjective Global Assessment (PG-SGA) was utilized for assessment, with Acute Gastrointestinal Injury (AGI) or AGI Ultrasonography (AGIUS) additionally for critically ill patients. The type and etiology of malnutrition were identified by comprehensive evaluation. Therefore, a strategy of “rapid screening for high-risk individuals → precise assessment of malnutrition severity → selection of treatment protocols” was established. Based on this, BC patients underwent stratified management (nutritional risk and no nutritional risk).

### 4.2 Focus of nutritional management across treatment methods and nutritional statuses

The focus of nutritional management varied across treatment methods and nutritional statuses. BC patients were assigned to four groups based on treatment methods (whether RC was performed) and nutritional risk (Yes/No), with different focuses each: (1) No nutritional risk + no RC (mostly non-muscle invasive or advanced/metastatic): primarily dietary counseling and nutrition education to maintain or improve nutritional status. (2) No nutritional risk + RC: In addition to dietary counseling and education, a perioperative Enhanced Recovery After Surgery (ERAS) protocol to facilitate postoperative rehabilitation. (3) Nutritional risk + no RC: In addition to dietary counseling and education, individualized nutrition support (including ONS and immune nutrients) as early as possible to maintain body weight, relieve treatment-related toxicity, and delay cachexia. (4) Nutritional risk + RC: preoperative prehabilitation and carbohydrate loading, early postoperative oral feeding, and stepwise nutritional transition, with long-term surveillance of complications related to ileal urinary diversion (e.g., vitamin B12 deficiency, metabolic acidosis, and mucus obstruction) to reduce complications, promote gastrointestinal functional recovery, and shorten hospital stays.

### 4.3 Interpretation and restatement of specific indicators

#### 4.3.1 Interpretation of three-tier diagnosis indicators

##### 4.3.1.1 First-level nutritional screening

Bladder cancer patients (especially the older ones and those with chronic hematuria) often have developed malnutrition at the time of diagnosis. Screening should be conducted “immediately upon diagnosis” (28) and “within 24 h post-admission for hospitalized patients” (29). For those identified as at no risk in the primary screening, a secondary screening is recommended 1 week later to prevent missed screening due to nutritional deterioration caused by examinations and treatment side effects during hospitalization. NRS2002 covers three dimensions (nutritional status impairment, disease severity, and age), and a total score ≥ 3 indicates nutritional risk. Although Malnutrition Universal Screening Tool (MUST) and Mini Nutritional Assessment-Short Form (MNA-SF) have advantages and disadvantages, and MNA-SF was recommended for older patients in the consultation letter, NRS2002 remained the preferred choice after discussion. It simultaneously assesses disease and nutritional status, and has a strong evidence base, wide global validation, and high sensitivity and specificity in cancer patients, so NRS2002 has been recommended by multiple guidelines (29). Additionally, m-NUTRIC can be added for critical illness screening for patients with severe infection or multi-organ dysfunction, with a score ≥ 5 as the threshold for severe nutritional risk (29).

##### 4.3.1.2 Second-level nutritional assessment

Patient-Generated Subjective Global Assessment is preferred for BC patients positive in nutritional screening, as it is specifically designed for cancer patients, covers dimensions such as weight, feeding, symptoms, function, and metabolic needs, and is more sensitive than general tools. Meanwhile, Global Leadership Initiative on Malnutrition (GLIM) criteria are recommended for malnutrition diagnosis, which include three phenotypic criteria and two etiological criteria, and have a clear structure, making it suitable for Chinese patients. For critically ill patients, AGI (four grades based on clinical presentation; higher grades indicate poorer prognosis) or AGIUS may be utilized to dynamically assess gastrointestinal function. These tools may be selected or combined based on clinical conditions to precisely guide nutritional planning (29).

##### 4.3.1.3 Third-level comprehensive evaluation

The mechanisms of malnutrition in cancer patients are diverse, and individualized interventions are required: Anti-inflammatory nutrition (e.g., ω-3 fatty acids) is required for patients with high inflammation status, ONS or tube feeding for those with inadequate intake, and protein intake and resistance training for those with muscle wasting. The third-level comprehensive evaluation guides individualized treatment based on energy consumption, stress level, inflammatory burden, and metabolic status. Given the difficult operation and high cost of TNF-α, IL-1, and IBI tests in primary hospitals, the expert panel recommended four-level evaluation as an “ideal recommendation” for high-level medical centers or research settings. In routine clinical practice, CRP, albumin, prealbumin, and clinical NIS scores can serve as substitutes, with a note stating that “selected based on the capabilities of the healthcare institution.”

#### 4.3.2 Interpretation of indicators for nutrition support therapy principles

##### 4.3.2.1 Stepwise approach and 60%/50% threshold switching

A stepwise approach should be followed for nutrition support, starting with dietary education and progressing step by step (ONS → TEN → PPN → TPN). Nutrition support should escalate when the lower level cannot meet ≥ 60% of the target nutritional needs, or deescalate if the lower level can meet ≥ 50% of the nutritional needs to avoid unnecessary high-level interventions. Based on the typical patterns of gastrointestinal functional recovery, the observation window is 3–5 d for general patients and 2–3 d for critically ill patients, balancing thorough observation and adequate feeding. Excessive nutrition support (especially parenteral nutrition) will increase the risk of infection, liver injury, and costs (27).

#### 4.3.3 Interpretation of intervention indicators at different treatment stages

##### 4.3.3.1 Preoperative prehabilitation and immunonutrition

Preoperative prehabilitation (2–6 weeks of multimodal intervention) is a core strategy of ERAS. Through the synergy of physical exercise, nutrition support, and psychological intervention, it enhances physiological reserves and reduces postoperative complications (29, 30). Immunonutrition (arginine, nucleotides, and ω-3 fatty acids) is preferred in the perioperative period in RC, as it enhances immunity, relieves inflammation, and facilitates postoperative recovery (31, 32). Enteral nutrition (oral or tube feeding) should be the first choice to maintain the intestinal barrier and improve immunity, as well as parenteral nutrition only when necessary (33). Experts indicate that immunonutrition should not be routinely given to all patients undergoing BC surgery, but should be selectively applied to: (1) those with moderate or severe malnutrition preoperatively, and (2) older patients with slow postoperative recovery or those with multiple comorbidities. Immunonutrition is not mandatory for patients with generally good nutritional status undergoing RC or transurethral resection of bladder.

##### 4.3.3.2 Preoperative fasting duration

To reduce the inhalation risk and enhance patient comfort, a preoperative fasting duration of 2 h for clear liquids and 6 h for solid foods is recommended in the ERAS framework (13). Carbohydrate loading can mitigate glucose fluctuation caused by insulin resistance, relieve postoperative hunger, vomiting, and nausea, and facilitate gastrointestinal recovery (34). Therefore, moderate intake of carbohydrate-rich beverages is recommended preoperatively for BC with no diabetes or well-controlled blood glucose, regardless of nutritional risk status (35). However, due to low clinical availability, medical workers should communicate with anesthesiologists to improve awareness and adoption rates.

##### 4.3.3.3 Measures to facilitate postoperative gastrointestinal recovery

Opioids, acting on μ-opioid receptors in the intestine, restrain gastrointestinal motility (36), so guidelines recommend reducing opioid use for postoperative gastrointestinal recovery in RC (30). Routine preoperative bowel preparation is not recommended (17, 30), but adjustments may be made based on China’s clinical practices and individual circumstances. Non-pharmacological interventions such as chewing gum and TCM acupoints (e.g., Zusanli) are recommended, as they are safe and low-cost, and align with the ERAS philosophy. When using Chinese herbal decoctions, the principles of syndrome differentiation should be strictly followed, with precise herbal selection and close attention to contraindications and drug safety (37). First, TCM use must be based on syndrome differentiation, avoiding a mechanistic “disease-label” approach. For complex cases, the priorities between root and branch should be weighed to avoid inappropriate use of warm-drying or cold-cooling agents. Second, contraindications should be observed. Patients with bleeding tendencies or coagulation disorders should not be given blood-activating and stasis-removing agents. Preparations metabolized by the liver/kidneys or containing heavy metals should be avoided for patients with hepatic or renal insufficiency. Long-term users of TCM should undergo liver and kidney function monitoring every 3–6 months. Third, during combined use of Chinese and Western medicines, coagulation function and other key indicators should be closely monitored for potential drug interactions. Fourth, a detailed inquiry regarding allergy/bleeding history, liver/kidney function, and concomitant medications should be conducted before treatment. Regular monitoring of coagulation, platelet count, and liver/kidney function should be performed during treatment. If abnormalities such as ecchymosis, bleeding, melena, palpitations, or dyspnea occur, the medication should be discontinued immediately, and clinical evaluation should be performed. Alvimopan is excluded from the recommendations due to its limited availability, high cost, and exclusion from medical insurance coverage in China.

##### 4.3.3.4 Early postoperative feeding and mobilization

Early postoperative oral feeding is encouraged for RC patients with or without nutritional risk, as it is safe and promotes recovery. However, for patients with nutritional risk, a high carbohydrate-to-fat ratio should be avoided during the postoperative acute period to prevent exacerbating glycometabolic disorders. Nevertheless, early feeding still raises a concern about increased risk of postoperative intestinal obstruction and stomal leak (38). This conventional view hinders evidence-based practice and reflects the gap between clinical practice and evidence. Therefore, efforts should be made to dispel misconceptions, strengthen education for patients and nurses, and popularize early oral feeding. Future studies should clarify the optimal indications and treatment timing for RC. Enteral or parenteral nutrition support should be provided for patients with nutritional risk as necessary. Early mobilization is central to ERAS and helps restore gastrointestinal function and lower the risk of complications (39). Since RC patients are predominantly older with comorbidities and face a heavy postoperative burden (40), mobilization initiated within 24 h postoperatively is recommended, gradually progressing from bed exercises to sitting, standing, and independent walking (41).

##### 4.3.3.5 Application of the nutritional framework in hospitals with limited nutritional resources

In clinical practice, the actual circumstances of hospitals with limited nutritional resources should be taken into consideration. Healthcare providers may use NRS2002, a simple nutritional screening tool, for nutritional screening at the time of admission, and employ PG-SGA for nutritional assessment. Based on the patient’s nutritional status, stratified interventions can then be implemented. Additionally, a nutritional management module may be integrated into the electronic medical record system to facilitate outcome monitoring.

### 4.4 Strengths and limitations

#### 4.4.1 Strengths

This study fully covered the entire process of nutritional management of BC, forming a closed-loop evidence chain from team formation to follow-up, from screening to three-tier diagnosis, and from different nutritional risks to the presence or absence of RC. TCM interventions (e.g., auricular therapy and acupoint massage) were integrated on an evidence-based foundation to help alleviate anxiety and nausea and promote gastrointestinal recovery (42). We, for the first time, clearly distinguished whether RC was performed and further stratified patients based on the nutritional risk. Cutting-edge concepts and China’s realities were also taken into account, such as incorporating high-level evidence on three-tier diagnosis and immunonutrition, and localized adjustments were made to address issues like the limited availability of alvimopan, thereby achieving a balance among efficacy, feasibility, and acceptability.

#### 4.4.2 Limitations

The following limitations are worth noting: BC-specific studies were insufficient, with most evidence derived from other cancers or the general population. Health economic evaluations were lacking, making it difficult to assess the cost-effectiveness of interventions. Some indicator tests (e.g., inflammatory burden index, cytokine testing) are less feasible in primary hospitals. TCM techniques face challenges in cultural acceptance and applicability in Western nursing settings. The evidence base is weak, lacking high-level evidence due to poor quality of the literature and non-unique entries, with no RCTs or systematic reviews incorporated.

In this study, the Kendall’s coefficients of concordance for the two rounds of expert consultation were at a moderately low level (*P* < 0.001), indicating that the consistency among expert opinions was statistically significant. Following the two rounds of expert consultation, the Kendall’s coefficient of concordance increased from 0.250 to 0.313, suggesting that disagreement among experts diminished and opinions tended to converge. These results also indicate that the moderate values of Kendall’s coefficients of concordance did not affect the overall direction of the study. The possible reasons are as follows: (1) a large number of evidence summaries; (2) the involvement of experts of different specialties; and (3) substantial cognitive discrepancies regarding certain evidence items (e.g., TCM techniques), which reflect academic controversy rather than methodological issues.

## 5 Conclusion

This study created a whole-course nutritional management system for BC, and yielded the following clinical recommendations: (1) Clinical teams should prioritize the foundational process of “screening-assessment-intervention-surveillance” and gradually implement refined management. (2) We should establish multidisciplinary nutrition support teams for routine collaboration. (3) We should strengthen the nutritional self-management capabilities of patients and caregivers (e.g., recognizing NIS symptoms and monitoring body weight), extending these capabilities to the home setting. In the future, BC-specific, multicenter, large-sample original studies are required to validate immunonutrition, TCM, and individualized ONS for cost-effectiveness and efficacy. Nurses’ evidence-based practice capabilities and multidisciplinary collaboration should be enhanced, and culturally adapted TCM strategies and nutrition care models integrating Eastern and Western approaches should be explored.

## Data Availability

The original contributions presented in this study are included in this article/Supplementary material, further inquiries can be directed to the corresponding author.
