# Supplementary material for: Summary of the best evidence for whole-course nutritional management of bladder cancer
Source: Front Nutr. 2026 Aug 13;13:1916117. doi: 10.3389/fnut.2026.1916117 (PMC13518117; doi:10.3389/fnut.2026.1916117)
Supplement: Supplementary file 1 [file Table_1.docx]

**Supplementary materials**

**Appendix A.** Search Strategy

**1.BMJ Search Strategy**

Search date: 2025.12.15

Search term: Bladder cancer

Downloaded 1 article

**2.CINAHL Search Strategy**

CINAHL Search Strategy.(2025-12-12)

| No. | Search Expression | Results |
| --- | --- | --- |
| S1 | SU (MH "Bladder Neoplasms") OR TI (“bladder neoplasm” or “bladder tumor” or “bladder cancer” or “bladder malignancy” or “bladder tumorigenesis” or “bladder neoplasia ” or “carcinomatous bladder” or “neoplastic bladder” or “vesical cancer” or “vesical tumor” or “cancer of bladder” or “malignancy of the bladder” or “neoplasia of the bladder” or “neoplasm of the bladder” or “tumor of the bladder” or “ malignant tumor of urinary bladder”) OR AB (“bladder neoplasm” or “bladder tumor” or “bladder cancer” or “bladder malignancy” or “bladder tumorigenesis” or “bladder neoplasia ” or “carcinomatous bladder” or “neoplastic bladder” or “vesical cancer” or “vesical tumor” or “cancer of bladder” or “malignancy of the bladder” or “neoplasia of the bladder” or “neoplasm of the bladder” or “tumor of the bladder” or “ malignant tumor of urinary bladder”) | 8829 |
| S2 | MH "Transurethral Resection of Bladder" OR (“transurethral resection of bladder”or “bladder transurethral resection ” or “bladder excision” or “bladder extirpation” or “bladder resection” or “pericystectomy” or “urinary bladder removal”) OR (“transurethral resection of bladder”or “bladder transurethral resection ” or “bladder excision” or “bladder extirpation” or “bladder resection” or “pericystectomy” or “urinary bladder removal”) | 536 |
| S3 | SU (MH "Cystectomy") OR TI (cystectomy or “radical cystectomy” or “partial cystectomy” or “complete cystectomy” or “total cystectomy”) OR AB (cystectomy or “radical cystectomy” or “partial cystectomy” or “complete cystectomy” or “total cystectomy”) | 3361 |
| S4 | nutrition* or “nutritional management” or “nutritional support” or “nutritional risk” or “nutritional assessment” or “perioperative nutrition” or “nutritional education” or “nutrition therapy” or diet | 331437 |
| S5 | guideline or “expert consensus” or “expert recommendation” or “evidence summary” or “systematic review” or “randomized controlled trial” | 534659 |
| S6 | S1 OR S2 OR S3 | 10755 |
| S7 | S4 AND S5 AND S6 | 31 |

EBSCO-Search-history-metadata-12_12_2025

| S# | Search Name | Search Description | Query (user-entered) | Query (expanded/display term) | Search run Date and Time | Results (count) | Search Mode | Expander(s) | Interface | Database(s) |
| --- | --- | --- | --- | --- | --- | --- | --- | --- | --- | --- |
| S7 |  |  | S4 AND S5 AND S6 | (nutrition* or “nutritional management” or “nutritional support” or “nutritional risk” or “nutritional assessment” or “perioperative nutrition” or “nutritional education” or “nutrition therapy” or diet) AND (guideline or “expert consensus” or “expert recommendation” or “evidence summary” or “systematic review” or “randomized controlled trial”) AND ((SU (MH "Bladder Neoplasms") OR TI (“bladder neoplasm” or “bladder tumor” or “bladder cancer” or “bladder malignancy” or “bladder tumorigenesis” or “bladder neoplasia ” or “carcinomatous bladder” or “neoplastic bladder” or “vesical cancer” or “vesical tumor” or “cancer of bladder” or “malignancy of the bladder” or “neoplasia of the bladder” or “neoplasm of the bladder” or “tumor of the bladder” or “ malignant tumor of urinary bladder”) OR AB (“bladder neoplasm” or “bladder tumor” or “bladder cancer” or “bladder malignancy” or “bladder tumorigenesis” or “bladder neoplasia ” or “carcinomatous bladder” or “neoplastic bladder” or “vesical cancer” or “vesical tumor” or “cancer of bladder” or “malignancy of the bladder” or “neoplasia of the bladder” or “neoplasm of the bladder” or “tumor of the bladder” or “ malignant tumor of urinary bladder”)) OR (MH "Transurethral Resection of Bladder" OR (“transurethral resection of bladder”or “bladder transurethral resection ” or “bladder excision” or “bladder extirpation” or “bladder resection” or “pericystectomy” or “urinary bladder removal”) OR (“transurethral resection of bladder”or “bladder transurethral resection ” or “bladder excision” or “bladder extirpation” or “bladder resection” or “pericystectomy” or “urinary bladder removal”)) OR (SU (MH "Cystectomy") OR TI (cystectomy or “radical cystectomy” or “partial cystectomy” or “complete cystectomy” or “total cystectomy”) OR AB (cystectomy or “radical cystectomy” or “partial cystectomy” or “complete cystectomy” or “total cystectomy”))) | 2025-12-12T03:29:38.587Z | 31 | Proximity | Application of equivalent subjects | EBSCOhost Research Databases | CINAHL Complete |
| S6 |  |  | S1 OR S2 OR S3 | (SU (MH "Bladder Neoplasms") OR TI (“bladder neoplasm” or “bladder tumor” or “bladder cancer” or “bladder malignancy” or “bladder tumorigenesis” or “bladder neoplasia ” or “carcinomatous bladder” or “neoplastic bladder” or “vesical cancer” or “vesical tumor” or “cancer of bladder” or “malignancy of the bladder” or “neoplasia of the bladder” or “neoplasm of the bladder” or “tumor of the bladder” or “ malignant tumor of urinary bladder”) OR AB (“bladder neoplasm” or “bladder tumor” or “bladder cancer” or “bladder malignancy” or “bladder tumorigenesis” or “bladder neoplasia ” or “carcinomatous bladder” or “neoplastic bladder” or “vesical cancer” or “vesical tumor” or “cancer of bladder” or “malignancy of the bladder” or “neoplasia of the bladder” or “neoplasm of the bladder” or “tumor of the bladder” or “ malignant tumor of urinary bladder”)) OR (MH "Transurethral Resection of Bladder" OR (“transurethral resection of bladder”or “bladder transurethral resection ” or “bladder excision” or “bladder extirpation” or “bladder resection” or “pericystectomy” or “urinary bladder removal”) OR (“transurethral resection of bladder”or “bladder transurethral resection ” or “bladder excision” or “bladder extirpation” or “bladder resection” or “pericystectomy” or “urinary bladder removal”)) OR (SU (MH "Cystectomy") OR TI (cystectomy or “radical cystectomy” or “partial cystectomy” or “complete cystectomy” or “total cystectomy”) OR AB (cystectomy or “radical cystectomy” or “partial cystectomy” or “complete cystectomy” or “total cystectomy”)) | 2025-12-12T03:29:10.213Z | 10755 | Proximity | Application of equivalent subjects | EBSCOhost Research Databases | CINAHL Complete |
| S5 |  |  | guideline or “expert consensus” or “expert recommendation” or “evidence summary” or “systematic review” or “randomized controlled trial” | guideline or “expert consensus” or “expert recommendation” or “evidence summary” or “systematic review” or “randomized controlled trial” | 2025-12-12T03:28:35.682Z | 534659 | Proximity | Application of equivalent subjects | EBSCOhost Research Databases | CINAHL Complete |
| S4 |  |  | nutrition* or “nutritional management” or “nutritional support” or “nutritional risk” or “nutritional assessment” or “perioperative nutrition” or “nutritional education” or “nutrition therapy” or diet | nutrition* or “nutritional management” or “nutritional support” or “nutritional risk” or “nutritional assessment” or “perioperative nutrition” or “nutritional education” or “nutrition therapy” or diet | 2025-12-12T03:28:12.941Z | 331437 | Proximity | Application of equivalent subjects | EBSCOhost Research Databases | CINAHL Complete |
| S3 |  |  | SU (MH "Cystectomy") OR TI (cystectomy or “radical cystectomy” or “partial cystectomy” or “complete cystectomy” or “total cystectomy”) OR AB (cystectomy or “radical cystectomy” or “partial cystectomy” or “complete cystectomy” or “total cystectomy”) | SU (MH "Cystectomy") OR TI (cystectomy or “radical cystectomy” or “partial cystectomy” or “complete cystectomy” or “total cystectomy”) OR AB (cystectomy or “radical cystectomy” or “partial cystectomy” or “complete cystectomy” or “total cystectomy”) | 2025-12-12T03:27:50.788Z | 3361 | Proximity | Application of equivalent subjects | EBSCOhost Research Databases | CINAHL Complete |
| S2 |  |  | MH "Transurethral Resection of Bladder" OR (“transurethral resection of bladder”or “bladder transurethral resection ” or “bladder excision” or “bladder extirpation” or “bladder resection” or “pericystectomy” or “urinary bladder removal”) OR (“transurethral resection of bladder”or “bladder transurethral resection ” or “bladder excision” or “bladder extirpation” or “bladder resection” or “pericystectomy” or “urinary bladder removal”) | MH "Transurethral Resection of Bladder" OR (“transurethral resection of bladder”or “bladder transurethral resection ” or “bladder excision” or “bladder extirpation” or “bladder resection” or “pericystectomy” or “urinary bladder removal”) OR (“transurethral resection of bladder”or “bladder transurethral resection ” or “bladder excision” or “bladder extirpation” or “bladder resection” or “pericystectomy” or “urinary bladder removal”) | 2025-12-12T03:26:41.312Z | 536 | Proximity | Application of equivalent subjects | EBSCOhost Research Databases | CINAHL Complete |
| S1 |  |  | SU (MH "Bladder Neoplasms") OR TI (“bladder neoplasm” or “bladder tumor” or “bladder cancer” or “bladder malignancy” or “bladder tumorigenesis” or “bladder neoplasia ” or “carcinomatous bladder” or “neoplastic bladder” or “vesical cancer” or “vesical tumor” or “cancer of bladder” or “malignancy of the bladder” or “neoplasia of the bladder” or “neoplasm of the bladder” or “tumor of the bladder” or “ malignant tumor of urinary bladder”) OR AB (“bladder neoplasm” or “bladder tumor” or “bladder cancer” or “bladder malignancy” or “bladder tumorigenesis” or “bladder neoplasia ” or “carcinomatous bladder” or “neoplastic bladder” or “vesical cancer” or “vesical tumor” or “cancer of bladder” or “malignancy of the bladder” or “neoplasia of the bladder” or “neoplasm of the bladder” or “tumor of the bladder” or “ malignant tumor of urinary bladder”) | SU (MH "Bladder Neoplasms") OR TI (“bladder neoplasm” or “bladder tumor” or “bladder cancer” or “bladder malignancy” or “bladder tumorigenesis” or “bladder neoplasia ” or “carcinomatous bladder” or “neoplastic bladder” or “vesical cancer” or “vesical tumor” or “cancer of bladder” or “malignancy of the bladder” or “neoplasia of the bladder” or “neoplasm of the bladder” or “tumor of the bladder” or “ malignant tumor of urinary bladder”) OR AB (“bladder neoplasm” or “bladder tumor” or “bladder cancer” or “bladder malignancy” or “bladder tumorigenesis” or “bladder neoplasia ” or “carcinomatous bladder” or “neoplastic bladder” or “vesical cancer” or “vesical tumor” or “cancer of bladder” or “malignancy of the bladder” or “neoplasia of the bladder” or “neoplasm of the bladder” or “tumor of the bladder” or “ malignant tumor of urinary bladder”) | 2025-12-12T03:25:21.243Z | 8829 | Proximity | Application of equivalent subjects | EBSCOhost Research Databases | CINAHL Complete |

**3.CNKI Search Strategy**

**CNKI Search Strategy.(**Publication Date: –**2025.12)**

| No. | Search Expression | Results |
| --- | --- | --- |
| #1 | (Title, Abstract, Keywords: Bladder cancer + Bladder tumor + Transurethral resection of bladder + Radical cystectomy + Bladder resection + Urinary diversion + Urinary tract diversion + Vesicostomy + Ureterostomy (Exact)) AND (Title, Abstract, Keywords: Nutrition + Nutritional support + Nutritional management + Nutritional strategy + Enteral nutrition + Parenteral nutrition + Malnutrition + Nutritional risk + Nutritional assessment + Perioperative nutrition (Exact)) | **187** |

**4.COCHRANE Search Strategy**

Cochrane Search Strategy.

| No. | Search Expression | Results |
| --- | --- | --- |
| #1 | MeSH descriptor: [Urinary Bladder Neoplasms] explode all trees | 2517 |
| #2 | bladder neoplasm" or "bladder tumor" or “bladder cancer" or *bladder malignancy" or *bladder tumorigenesis" or "bladder neoplasia " or "carcinomatous bladder" or  neoplastic bladder" or "vesical cancer" or "vesical tumor" or "cancer of bladder" or "malignancy of the bladder" or "neoplasia of the bladder" or "neoplasm of the  bladder" or "tumor of the bladder* or " malignant tumor of urinary bladder" | 4718 |
| #3 | #1 OR #2 | 5391 |
| #4 | MeSH descriptor: [Transurethral Resection of Bladder] explode all trees | 24 |
| #5 | "bladder transurethral resection " or "bladder excision" or "bladder extirpation" or "bladder resection" or “pericystectomy" or "urinary bladder removal" | 53 |
| #6 | #4 OR #5 | 77 |
| #7 | MeSH descriptor: [Cystectomy] explode altrees | 608 |
| #8 | "radical cystectomy" or *partial cystectomy" or "complete cystectomy" or total cystectomy" | 1347 |
| #9 | #7 OR #8 | 1596 |
| #10 | #3 OR #6 OR #9 | 5772 |
| #11 | nutrition* or "nutritional management" or "nutritional support" or "nutritional risk" or “nutritional assessment" or "perioperative nutrition" or *nutritional education" or  "nutrition therapy" or diet | 151014 |
| #12 | guideline or "expert consensus" or "expert recommendation" or  "evidence summary" or "systematic review" or "randomized controlled trial" | 774488 |
| #13 | #10 AND #11 AND #12 | 95 |

Cochrane

**5.** **EMBASE Search Strategy**

Embase Search Strategy(Embase session results (10 Dec 2025)

| No. | Search Expression | Results |
| --- | --- | --- |
| #1 | 'bladder tumor'/exp | 129945 |
| #2 | 'bladder tumor' OR 'bladder neoplasm' OR 'bladder cancer' OR 'bladder malignancy' OR 'bladder tumorigenesis' OR 'bladder neoplasia' OR 'carcinomatous bladder' OR 'neoplastic bladder' OR 'vesical cancer' OR 'vesical tumor' OR 'cancer of bladder' OR 'malignancy of the bladder' OR 'neoplasia of the bladder' OR 'neoplasm of the bladder' OR 'tumor of the bladder' OR 'malignant tumor of urinary bladder' | 124390 |
| #3 | #1 OR #2 | 139124 |
| #4 | 'cystectomy'/exp | 44488 |
| #5 | cystectomy OR 'bladder transurethral resection' OR 'bladder excision' OR 'bladder extirpation' OR 'bladder resection' OR 'pericystectomy' OR 'urinary bladder removal' | 49253 |
| #6 | #4 OR #5 | 50802 |
| #7 | 'radical cystectomy'/exp | 5697 |
| #8 | 'radical cystectomy' OR 'partial cystectomy' OR 'complete cystectomy' OR 'total cystectomy' | 25219 |
| #9 | #7 OR #8 | 25219 |
| #10 | #3 OR #6 OR #9 | 159770 |
| #11 | nutrition* OR 'nutritional management' OR 'nutritional support' OR 'nutritional risk' OR 'nutritional assessment' OR 'perioperative nutrition' OR 'nutritional education' OR 'nutrition therapy' OR diet | 2067837 |
| #12 | guideline OR 'expert consensus' OR 'expert recommendation' OR 'evidence summary' OR 'systematic review' OR 'randomized controlled trial' | 2653399 |
| #13 | #10 AND #11 AND #12 | 566 |

Queries

**6.Guidelines**

Search Strategy for Guidelines(10)

| **Database** | **Search Terms** | **Results** |
| --- | --- | --- |
| RNAO | bladder cancer | 1 |
| GIN | bladder cancer | 12 |
| NCCN | bladder cancer | 2 |
| AHRQ | bladder cancer | 4 |
| NICE | bladder cancer/  stoma | 4 |
| Yimaitong | bladder cancer | 1 |
| WHO | bladder cancer | 0 |
| SIGN | bladder cancer | 0 |
| NZGG | bladder cancer | 0 |
| NHMRC | bladder cancer | 0 |

**7.JBI Search Strategy**

JBI Search Strategy.

JBI EBP Database <Current to November 12, 2025>

| No. | Search Expression | Results |
| --- | --- | --- |
| 1 | ("bladder neoplasm" or "bladder tumor" or "bladder cancer" or "bladder malignancy" or "bladder tumorigenesis" or "bladder neoplasia " or "carcinomatous bladder" or "neoplastic bladder" or "vesical cancer" or "vesical tumor" or "cancer of bladder" or "malignancy of the bladder" or "neoplasia of the bladder" or "neoplasm of the bladder" or "tumor of the bladder" or " malignant tumor of urinary bladder" or "transurethral resection of bladder" or "bladder excision" or "bladder extirpation" or "bladder resection" or "pericystectomy" or cystectomy).mp. [mp=text, heading word, subject area node word, title] | 21 |
| 2 | (nutrition* or "nutritional management" or "nutritional support" or "nutritional risk" or "nutritional assessment" or "perioperative nutrition" or "nutritional education" or "nutrition therapy" or diet).mp. [mp=text, heading word, subject area node word, title] | 1426 |
| 3 | (guideline or "expert consensus" or "expert recommendation" or "evidence summary" or "systematic review" or "randomized controlled trial").mp. [mp=text, heading word, subject area node word, title] | 7907 |
| 4 | 1 and 2 and 3 | 7 |

**8.Professional Organizations**

Search Strategy for Expert Consensus(14)

| Database | Search Terms | Results |
| --- | --- | --- |
| WOCN | bladder cancer | 1 |
| WCET | bladder cancer | 1 |
| EAU | bladder cancer | 2 |
| UOAA | bladder cancer | 4 |
| CUA | bladder cancer | 6 |
| ESPEN | bladder cancer | 6 |
| AUA | “bladder cancer nutrition” | 11 |
| SIU | “bladder cancer”(abstract)and nutrition(abstract) | 16 |
| Urology Branch of Chinese Medical Association | bladder cancer/ stoma and nutrition | 4 |
| Cancer Nutrition Management Branch of Chinese Nutrition Society | bladder cancer | 3 |
| CAET | bladder cancer | 0 |
| ACP | bladder cancer | 0 |
| ASCRS | bladder cancer | 0 |
| CUSA | bladder cancer | 0 |

**9.PUBMED Search Strategy**

PubMed Search Strategy

| No. | Search Expression | Results |
| --- | --- | --- |
| #1 | "Urinary Bladder Neoplasms"[Mesh] | 68960 |
| #2 | "bladder neoplasm"[Title/Abstract] OR "bladder tumor"[Title/Abstract] OR "bladder cancer"[Title/Abstract] OR "bladder malignancy"[Title/Abstract] OR "bladder tumorigenesis"[Title/Abstract] OR "bladder neoplasia "[Title/Abstract] OR "carcinomatous bladder"[Title/Abstract] OR "neoplastic bladder"[Title/Abstract] OR "vesical cancer"[Title/Abstract] OR "vesical tumor"[Title/Abstract] OR "cancer of bladder"[Title/Abstract] OR "malignancy of the bladder"[Title/Abstract] OR "neoplasia of the bladder"[Title/Abstract] OR "neoplasm of the bladder"[Title/Abstract] OR "tumor of the bladder"[Title/Abstract] OR " malignant tumor of urinary bladder"[Title/Abstract] | 55523 |
| #3 | #1 OR #2 | 86079 |
| #4 | "Transurethral Resection of Bladder" [Mesh] | 108 |
| #5 | "bladder transurethral resection "[Title/Abstract] OR "bladder excision"[Title/Abstract] OR "bladder extirpation"[Title/Abstract] OR "bladder resection"[Title/Abstract] OR "pericystectomy"[Title/Abstract] OR "urinary bladder removal"[Title/Abstract] | 752 |
| #6 | #4 OR #5 | 860 |
| #7 | Cystectomy [Mesh] | 12333 |
| #8 | "radical cystectomy"[Title/Abstract] OR "partial cystectomy"[Title/Abstract] OR "complete cystectomy"[Title/Abstract] OR "total cystectomy"[Title/Abstract] | 13749 |
| #9 | #7 OR #8 | 18497 |
| #10 | #3 OR #6 OR #9 | 90523 |
| #11 | (nutrition* or "nutritional management" or "nutritional support" or "nutritional risk" or "nutritional assessment" or "perioperative nutrition" or "nutritional education" or "nutrition therapy" or diet) | 1449369 |
| #12 | (guideline or "expert consensus" or "expert recommendation" or "evidence summary" or "systematic review" or "randomized controlled trial") | 1473327 |
| #13 | #10 AND #11 AND #12 | 143 |
| #14 | #13 from 1978 - 2025 | 140 |

PubMed Search History

| Search number | Query | Sort By | Filters | Search Details | Results | Time | Date |
| --- | --- | --- | --- | --- | --- | --- | --- |
| 14 | #13 |  | from 1978 - 2025 | (("Urinary Bladder Neoplasms"[MeSH Terms] OR ("bladder neoplasm"[Title/Abstract] OR "bladder tumor"[Title/Abstract] OR "bladder cancer"[Title/Abstract] OR "bladder malignancy"[Title/Abstract] OR "bladder tumorigenesis"[Title/Abstract] OR "bladder neoplasia"[Title/Abstract] OR "carcinomatous bladder"[Title/Abstract] OR "neoplastic bladder"[Title/Abstract] OR "vesical cancer"[Title/Abstract] OR "vesical tumor"[Title/Abstract] OR "cancer of bladder"[Title/Abstract] OR "neoplasm of the bladder"[Title/Abstract] OR "tumor of the bladder"[Title/Abstract] OR "malignant tumor of urinary bladder"[Title/Abstract]) OR ("Transurethral Resection of Bladder"[MeSH Terms] OR ("bladder transurethral resection"[Title/Abstract] OR "bladder excision"[Title/Abstract] OR "bladder extirpation"[Title/Abstract] OR "bladder resection"[Title/Abstract] OR "pericystectomy"[Title/Abstract])) OR ("cystectomy"[MeSH Terms] OR ("radical cystectomy"[Title/Abstract] OR "partial cystectomy"[Title/Abstract] OR "complete cystectomy"[Title/Abstract] OR "total cystectomy"[Title/Abstract]))) AND ("nutrition*"[All Fields] OR "nutritional management"[All Fields] OR "nutritional support"[All Fields] OR "nutritional risk"[All Fields] OR "nutritional assessment"[All Fields] OR "perioperative nutrition"[All Fields] OR "nutritional education"[All Fields] OR "nutrition therapy"[All Fields] OR ("diet"[MeSH Terms] OR "diet"[All Fields])) AND ("guideline"[Publication Type] OR "guidelines as topic"[MeSH Terms] OR "guideline"[All Fields] OR "expert consensus"[All Fields] OR "expert recommendation"[All Fields] OR "evidence summary"[All Fields] OR "systematic review"[All Fields] OR "randomized controlled trial"[All Fields])) AND (1978:2025[pdat]) | 140 | 20:40:51 | 2026/4/28 |
| 13 | #10 AND #11 AND #12 | | | ("Urinary Bladder Neoplasms"[MeSH Terms] OR ("bladder neoplasm"[Title/Abstract] OR "bladder tumor"[Title/Abstract] OR "bladder cancer"[Title/Abstract] OR "bladder malignancy"[Title/Abstract] OR "bladder tumorigenesis"[Title/Abstract] OR "bladder neoplasia"[Title/Abstract] OR "carcinomatous bladder"[Title/Abstract] OR "neoplastic bladder"[Title/Abstract] OR "vesical cancer"[Title/Abstract] OR "vesical tumor"[Title/Abstract] OR "cancer of bladder"[Title/Abstract] OR "neoplasm of the bladder"[Title/Abstract] OR "tumor of the bladder"[Title/Abstract] OR "malignant tumor of urinary bladder"[Title/Abstract]) OR ("Transurethral Resection of Bladder"[MeSH Terms] OR ("bladder transurethral resection"[Title/Abstract] OR "bladder excision"[Title/Abstract] OR "bladder extirpation"[Title/Abstract] OR "bladder resection"[Title/Abstract] OR "pericystectomy"[Title/Abstract])) OR ("cystectomy"[MeSH Terms] OR ("radical cystectomy"[Title/Abstract] OR "partial cystectomy"[Title/Abstract] OR "complete cystectomy"[Title/Abstract] OR "total cystectomy"[Title/Abstract]))) AND ("nutrition*"[All Fields] OR "nutritional management"[All Fields] OR "nutritional support"[All Fields] OR "nutritional risk"[All Fields] OR "nutritional assessment"[All Fields] OR "perioperative nutrition"[All Fields] OR "nutritional education"[All Fields] OR "nutrition therapy"[All Fields] OR ("diet"[MeSH Terms] OR "diet"[All Fields])) AND ("guideline"[Publication Type] OR "guidelines as topic"[MeSH Terms] OR "guideline"[All Fields] OR "expert consensus"[All Fields] OR "expert recommendation"[All Fields] OR "evidence summary"[All Fields] OR "systematic review"[All Fields] OR "randomized controlled trial"[All Fields]) | 143 | 20:40:41 | 2026/4/28 |
| 12 | (guideline or "expert consensus" or "expert recommendation" or "evidence summary" or "systematic review" or "randomized controlled trial") | | | "guideline"[Publication Type] OR "guidelines as topic"[MeSH Terms] OR "guideline"[All Fields] OR "expert consensus"[All Fields] OR "expert recommendation"[All Fields] OR "evidence summary"[All Fields] OR "systematic review"[All Fields] OR "randomized controlled trial"[All Fields] | 1,473,327 | 20:40:11 | 2026/4/28 |
| 11 | (nutrition* or "nutritional management" or "nutritional support" or "nutritional risk" or "nutritional assessment" or "perioperative nutrition" or "nutritional education" or "nutrition therapy" or diet) | | | "nutrition*"[All Fields] OR "nutritional management"[All Fields] OR "nutritional support"[All Fields] OR "nutritional risk"[All Fields] OR "nutritional assessment"[All Fields] OR "perioperative nutrition"[All Fields] OR "nutritional education"[All Fields] OR "nutrition therapy"[All Fields] OR "diet"[MeSH Terms] OR "diet"[All Fields] | 1,449,369 | 20:39:44 | 2026/4/28 |
| 10 | #3 OR #6 OR #9 | |  | "Urinary Bladder Neoplasms"[MeSH Terms] OR "bladder neoplasm"[Title/Abstract] OR "bladder tumor"[Title/Abstract] OR "bladder cancer"[Title/Abstract] OR "bladder malignancy"[Title/Abstract] OR "bladder tumorigenesis"[Title/Abstract] OR "bladder neoplasia"[Title/Abstract] OR "carcinomatous bladder"[Title/Abstract] OR "neoplastic bladder"[Title/Abstract] OR "vesical cancer"[Title/Abstract] OR "vesical tumor"[Title/Abstract] OR "cancer of bladder"[Title/Abstract] OR "neoplasm of the bladder"[Title/Abstract] OR "tumor of the bladder"[Title/Abstract] OR "malignant tumor of urinary bladder"[Title/Abstract] OR "Transurethral Resection of Bladder"[MeSH Terms] OR "bladder transurethral resection"[Title/Abstract] OR "bladder excision"[Title/Abstract] OR "bladder extirpation"[Title/Abstract] OR "bladder resection"[Title/Abstract] OR "pericystectomy"[Title/Abstract] OR "cystectomy"[MeSH Terms] OR "radical cystectomy"[Title/Abstract] OR "partial cystectomy"[Title/Abstract] OR "complete cystectomy"[Title/Abstract] OR "total cystectomy"[Title/Abstract] | 90,523 | 20:39:05 | 2026/4/28 |
| 9 | #7 OR #8 |  |  | "cystectomy"[MeSH Terms] OR "radical cystectomy"[Title/Abstract] OR "partial cystectomy"[Title/Abstract] OR "complete cystectomy"[Title/Abstract] OR "total cystectomy"[Title/Abstract] | 18,497 | 20:38:22 | 2026/4/28 |
| 8 | "radical cystectomy"[Title/Abstract] OR "partial cystectomy"[Title/Abstract] OR "complete cystectomy"[Title/Abstract] OR "total cystectomy"[Title/Abstract] | | | "radical cystectomy"[Title/Abstract] OR "partial cystectomy"[Title/Abstract] OR "complete cystectomy"[Title/Abstract] OR "total cystectomy"[Title/Abstract] | 13,749 | 20:37:42 | 2026/4/28 |
| 7 | Cystectomy [Mesh] | |  | "cystectomy"[MeSH Terms] | 12,333 | 20:36:42 | 2026/4/28 |
| 6 | #4 OR #5 |  |  | "Transurethral Resection of Bladder"[MeSH Terms] OR "bladder transurethral resection"[Title/Abstract] OR "bladder excision"[Title/Abstract] OR "bladder extirpation"[Title/Abstract] OR "bladder resection"[Title/Abstract] OR "pericystectomy"[Title/Abstract] | 860 | 20:36:22 | 2026/4/28 |
| 5 | "bladder transurethral resection "[Title/Abstract] OR "bladder excision"[Title/Abstract] OR "bladder extirpation"[Title/Abstract] OR "bladder resection"[Title/Abstract] OR "pericystectomy"[Title/Abstract] OR "urinary bladder removal"[Title/Abstract] | | | "bladder transurethral resection"[Title/Abstract] OR "bladder excision"[Title/Abstract] OR "bladder extirpation"[Title/Abstract] OR "bladder resection"[Title/Abstract] OR "pericystectomy"[Title/Abstract] | 752 | 20:35:59 | 2026/4/28 |
| 4 | "Transurethral Resection of Bladder" [Mesh] | | | "Transurethral Resection of Bladder"[MeSH Terms] | 108 | 20:35:32 | 2026/4/28 |
| 3 | #1 OR #2 |  |  | "Urinary Bladder Neoplasms"[MeSH Terms] OR "bladder neoplasm"[Title/Abstract] OR "bladder tumor"[Title/Abstract] OR "bladder cancer"[Title/Abstract] OR "bladder malignancy"[Title/Abstract] OR "bladder tumorigenesis"[Title/Abstract] OR "bladder neoplasia"[Title/Abstract] OR "carcinomatous bladder"[Title/Abstract] OR "neoplastic bladder"[Title/Abstract] OR "vesical cancer"[Title/Abstract] OR "vesical tumor"[Title/Abstract] OR "cancer of bladder"[Title/Abstract] OR "neoplasm of the bladder"[Title/Abstract] OR "tumor of the bladder"[Title/Abstract] OR "malignant tumor of urinary bladder"[Title/Abstract] | 86,079 | 20:34:58 | 2026/4/28 |
| 2 | "bladder neoplasm"[Title/Abstract] OR "bladder tumor"[Title/Abstract] OR "bladder cancer"[Title/Abstract] OR "bladder malignancy"[Title/Abstract] OR "bladder tumorigenesis"[Title/Abstract] OR "bladder neoplasia "[Title/Abstract] OR "carcinomatous bladder"[Title/Abstract] OR "neoplastic bladder"[Title/Abstract] OR "vesical cancer"[Title/Abstract] OR "vesical tumor"[Title/Abstract] OR "cancer of bladder"[Title/Abstract] OR "malignancy of the bladder"[Title/Abstract] OR "neoplasia of the bladder"[Title/Abstract] OR "neoplasm of the bladder"[Title/Abstract] OR "tumor of the bladder"[Title/Abstract] OR " malignant tumor of urinary bladder"[Title/Abstract] | | | "bladder neoplasm"[Title/Abstract] OR "bladder tumor"[Title/Abstract] OR "bladder cancer"[Title/Abstract] OR "bladder malignancy"[Title/Abstract] OR "bladder tumorigenesis"[Title/Abstract] OR "bladder neoplasia"[Title/Abstract] OR "carcinomatous bladder"[Title/Abstract] OR "neoplastic bladder"[Title/Abstract] OR "vesical cancer"[Title/Abstract] OR "vesical tumor"[Title/Abstract] OR "cancer of bladder"[Title/Abstract] OR "neoplasm of the bladder"[Title/Abstract] OR "tumor of the bladder"[Title/Abstract] OR "malignant tumor of urinary bladder"[Title/Abstract] | 55,523 | 20:34:31 | 2026/4/28 |
| 1 | "Urinary Bladder Neoplasms"[Mesh] | | | "Urinary Bladder Neoplasms"[MeSH Terms] | 68,960 | 20:30:27 | 2026/4/28 |

**10.Scopus Search Strategy**

Scopus Search Strategy.(Publication Date: –**2025.12.13**)

| No. | Search Expression | Results |
| --- | --- | --- |
| #1 | ( TITLE-ABS-KEY ( "bladder neoplasm" OR "bladder tumor" OR "bladder cancer" OR "bladder malignancy" OR "bladder tumorigenesis" OR "bladder neoplasia " OR "carcinomatous bladder" OR "neoplastic bladder" OR "vesical cancer" OR "vesical tumor" OR "cancer of bladder" OR "malignancy of the bladder" OR "neoplasia of the bladder" OR "neoplasm of the bladder" OR "tumor of the bladder" OR " malignant tumor of urinary bladder" OR "transurethral resection of bladder" OR "bladder excision" OR "bladder extirpation" OR "bladder resection" OR "pericystectomy" OR cystectomy ) AND TITLE-ABS-KEY ( nutrition* OR "nutritional management" OR "nutritional support" OR "nutritional risk" OR "nutritional assessment" OR "perioperative nutrition" OR "nutritional education" OR "nutrition therapy" OR diet ) AND TITLE-ABS-KEY ( guideline OR "expert consensus" OR "expert recommendation" OR "evidence summary" OR "systematic review" OR "randomized controlled trial" ) ) | **468** |

**11.Sinomed Search Strategy**

Sinomed Search Strategy.(Publication Date: –2025.12.15)

| No. | Search Expression | Results |
| --- | --- | --- |
| #1 | "Nutrition" [Common Field: Intelligence] OR "Nutritional Support" [Common Field: Intelligence] OR "Nutrition Management" [Common Field: Intelligence] OR "Nutritional Strategy" [Common Field: Intelligence] OR "Enteral Nutrition" [Common Field: Intelligence] OR "Parenteral Nutrition" [Common Field: Intelligence] OR "Malnutrition" [Common Field: Intelligence] OR "Nutritional Risk" [Common Field: Intelligence] OR "Nutritional Assessment" [Common Field: Intelligence] | 849724 |
| #2 | "Bladder Cancer" [Common Field: Intelligence] OR "Bladder Tumor" [Common Field: Intelligence] OR "Transurethral Resection of Bladder Tumor" [Common Field: Intelligence] OR "Radical Cystectomy" [Common Field: Intelligence] OR "Bladder Excision" [Common Field: Intelligence] OR "Urinary Diversion" [Common Field: Intelligence] OR "Ureterostomy" [Common Field: Intelligence] OR "Cystostomy" [Common Field: Intelligence] | 123071 |
| #3 | #1 AND#2 | 630 |

**12.UP T0 DATE Search Strategy**

UP TO DATE:

Search date: 2025.12.15

Search term: Bladder cancer

Downloaded 1 article

**13.Wanfang Search Strategy**

Wanfang Search Strategy.(Publication Date: –2025.12.15)

| No. | Search Expression | Results |
| --- | --- | --- |
| #1 | Title/Abstract/Keywords: (Bladder Cancer OR Bladder Tumor OR Transurethral Resection of Bladder Tumor OR Radical Cystectomy OR Bladder Excision OR Urinary Diversion OR Cystostomy OR Ureterostomy) AND Title/Abstract/Keywords: (Nutrition OR Nutritional Support OR Nutrition Management OR Nutritional Strategy OR Enteral Nutrition OR Parenteral Nutrition OR Malnutrition OR Nutritional Risk OR Nutritional Assessment OR Perioperative Nutrition) | 326 |

**14.WOS Search Strategy**

Web of Science Search Strategy.

| No. | Search Expression | Results |
| --- | --- | --- |
| #1 | (TI=(( “bladder neoplasm” or “bladder tumor” or “bladder cancer” or “bladder malignancy” or “bladder tumorigenesis” or “bladder neoplasia ” or “carcinomatous bladder” or “neoplastic bladder” or “vesical cancer” or “vesical tumor” or “cancer of bladder” or “malignancy of the bladder” or “neoplasia of the bladder” or “neoplasm of the bladder” or “tumor of the bladder” or “ malignant tumor of urinary bladder” or “transurethral resection of bladder” or “bladder excision” or “bladder extirpation” or “bladder resection” or “pericystectomy” or cystectomy ) )) OR AB=(( “bladder neoplasm” or “bladder tumor” or “bladder cancer” or “bladder malignancy” or “bladder tumorigenesis” or “bladder neoplasia ” or “carcinomatous bladder” or “neoplastic bladder” or “vesical cancer” or “vesical tumor” or “cancer of bladder” or “malignancy of the bladder” or “neoplasia of the bladder” or “neoplasm of the bladder” or “tumor of the bladder” or “ malignant tumor of urinary bladder” or “transurethral resection of bladder” or “bladder excision” or “bladder extirpation” or “bladder resection” or “pericystectomy” or cystectomy ) ) | 75514 |
| #2 | (TI=((nutrition* or “nutritional management” or “nutritional support” or “nutritional risk” or “nutritional assessment” or “perioperative nutrition” or “nutritional education” or “nutrition therapy” or diet) )) OR AB=((nutrition* or “nutritional management” or “nutritional support” or “nutritional risk” or “nutritional assessment” or “perioperative nutrition” or “nutritional education” or “nutrition therapy” or diet) ) | 1206080 |
| #3 | (TI=((guideline or “expert consensus” or “expert recommendation” or “evidence summary” or “systematic review” or “randomized controlled trial”))) OR AB=((guideline or “expert consensus” or “expert recommendation” or “evidence summary” or “systematic review” or “randomized controlled trial”)) | 1377916 |
| #4 | #1 AND #2 AND #3 | 85 |
| #5 | #1 AND #2 AND #3 and 2026 (Exclude – Publication Years) | 82 |

search-history

| Entitlements | # | Search Query | Database | Results | Date Run |
| --- | --- | --- | --- | --- | --- |
| - WOS.IC: 1993 to 2026 - WOS.CCR: 1985 to 2026 - WOS.SCI: 1945 to 2026 - WOS.AHCI: 1977 to 2026 - WOS.BHCI: 2005 to 2026 - WOS.BSCI: 2005 to 2026 - WOS.ESCI: 2005 to 2026 - WOS.ISTP: 1990 to 2026 - WOS.SSCI: 1977 to 2026 - WOS.ISSHP: 1990 to 2026 | 1 | (TI=(( “bladder neoplasm” or “bladder tumor” or “bladder cancer” or “bladder malignancy” or “bladder tumorigenesis” or “bladder neoplasia ” or “carcinomatous bladder” or “neoplastic bladder” or “vesical cancer” or “vesical tumor” or “cancer of bladder” or “malignancy of the bladder” or “neoplasia of the bladder” or “neoplasm of the bladder” or “tumor of the bladder” or “ malignant tumor of urinary bladder” or “transurethral resection of bladder” or “bladder excision” or “bladder extirpation” or “bladder resection” or “pericystectomy” or cystectomy ) )) OR AB=(( “bladder neoplasm” or “bladder tumor” or “bladder cancer” or “bladder malignancy” or “bladder tumorigenesis” or “bladder neoplasia ” or “carcinomatous bladder” or “neoplastic bladder” or “vesical cancer” or “vesical tumor” or “cancer of bladder” or “malignancy of the bladder” or “neoplasia of the bladder” or “neoplasm of the bladder” or “tumor of the bladder” or “ malignant tumor of urinary bladder” or “transurethral resection of bladder” or “bladder excision” or “bladder extirpation” or “bladder resection” or “pericystectomy” or cystectomy ) ) | Web of Science Core Collection | 75514 | Wed Apr 29 2026 09:11:46 GMT+0800 |
| - WOS.IC: 1993 to 2026 - WOS.CCR: 1985 to 2026 - WOS.SCI: 1945 to 2026 - WOS.AHCI: 1977 to 2026 - WOS.BHCI: 2005 to 2026 - WOS.BSCI: 2005 to 2026 - WOS.ESCI: 2005 to 2026 - WOS.ISTP: 1990 to 2026 - WOS.SSCI: 1977 to 2026 - WOS.ISSHP: 1990 to 2026 | 2 | (TI=((nutrition* or “nutritional management” or “nutritional support” or “nutritional risk” or “nutritional assessment” or “perioperative nutrition” or “nutritional education” or “nutrition therapy” or diet) )) OR AB=((nutrition* or “nutritional management” or “nutritional support” or “nutritional risk” or “nutritional assessment” or “perioperative nutrition” or “nutritional education” or “nutrition therapy” or diet) ) | Web of Science Core Collection | 1206080 | Wed Apr 29 2026 09:12:39 GMT+0800 |
| - WOS.IC: 1993 to 2026 - WOS.CCR: 1985 to 2026 - WOS.SCI: 1945 to 2026 - WOS.AHCI: 1977 to 2026 - WOS.BHCI: 2005 to 2026 - WOS.BSCI: 2005 to 2026 - WOS.ESCI: 2005 to 2026 - WOS.ISTP: 1990 to 2026 - WOS.SSCI: 1977 to 2026 - WOS.ISSHP: 1990 to 2026 | 3 | (TI=((guideline or “expert consensus” or “expert recommendation” or “evidence summary” or “systematic review” or “randomized controlled trial”))) OR AB=((guideline or “expert consensus” or “expert recommendation” or “evidence summary” or “systematic review” or “randomized controlled trial”)) | Web of Science Core Collection | 1377916 | Wed Apr 29 2026 09:13:20 GMT+0800 |
| - WOS.IC: 1993 to 2026 - WOS.CCR: 1985 to 2026 - WOS.SCI: 1945 to 2026 - WOS.AHCI: 1977 to 2026 - WOS.BHCI: 2005 to 2026 - WOS.BSCI: 2005 to 2026 - WOS.ESCI: 2005 to 2026 - WOS.ISTP: 1990 to 2026 - WOS.SSCI: 1977 to 2026 - WOS.ISSHP: 1990 to 2026 | 4 | #1 AND #2 AND #3 | Web of Science Core Collection | 85 | Wed Apr 29 2026 09:14:02 GMT+0800 |
| - WOS.IC: 1993 to 2026 - WOS.CCR: 1985 to 2026 - WOS.SCI: 1945 to 2026 - WOS.AHCI: 1977 to 2026 - WOS.BHCI: 2005 to 2026 - WOS.BSCI: 2005 to 2026 - WOS.ESCI: 2005 to 2026 - WOS.ISTP: 1990 to 2026 - WOS.SSCI: 1977 to 2026 - WOS.ISSHP: 1990 to 2026 | 5 | #1 AND #2 AND #3 and 2026 (Exclude – Publication Years) | Web of Science Core Collection | 82 | Wed Apr 29 2026 09:39:28 GMT+0800 |

**Appendix B.** Explanations of 135 articles deleted

| No. | Literature Title | Author (Year, Journal) | Primary Reason for Exclusion |
| --- | --- | --- | --- |
| 1 | Guideline for the assessment and management of gastrointestinal symptoms following colorectal surgery—A UEG/ESCP/EAES/ESPCG/ESPEN/ESNM/ESSO collaboration. Part II—Good practice guidance \| sequelae to benign diseases | Gielen, A. H. C et al.(2024,United European Gastroenterology Journal) | The research object does not match |
| 2 | Effect of a leucine-rich supplement in combination with nutrition and physical exercise in advanced cancer patients: A randomized controlled intervention trial | Storck, L. J(2020,Clinical Nutrition) | The research object does not match |
| 3 | ESPEN guideline on clinical nutrition and hydration in geriatrics | Volkert, D et al.(2019,Clin Nutr) | The research object does not match |
| 4 | Essential Japanese Guidelines for the Prevention of Perioperative Infections in the Urological Field: 2023 Edition |  | The research content does not match. |
| 5 | Nonintravesical Interventions for Preventing Intravesical Recurrence in Patients With Nonmuscle-Invasive Bladder Cancer: A Systematic Review and Meta-Analysis | Tsuboi, I et al.(2025,Genitourinary Cancer) | The research content does not match. |
| 6 | Prognostic role of prognostic nutritional index in patients with bladder cancer: a systematic review and meta-analysis | Sun, J et al.(2025,FRONTIERS IN ONCOLOGY) | The research content does not match. |
| 7 | Dietary mineral intake and risk of bladder cancer: a systematic review and meta-analysis | Zhong, X et al.(2025,EUROPEAN JOURNAL OF CANCER PREVENTION) | The research content does not match. |
| 8 | National Surgical Quality Improvement Program audit of contemporary perioperative care for radical cystectomy | Pfail, J et al.(2025,BJU INTERNATIONAL) | The research content does not match. |
| 9 | Construction and Empirical Study of a Perioperative Symptom Cluster Intervention Protocol for Bladder Cancer Patients Undergoing Urinary Diversion | Li Ling et al.(2025,Chinese Nursing Research) | The research content does not match. |
| 10 | Effect of Perioperative Immunonutrition on Early-Postoperative Complications in Patients Undergoing Radical Cystectomy for Bladder Cancer: A Case Series | Cianflone, F et al.(2025,Journal of Clinical Medicine) | The research content does not match. |
| 11 | ESPEN guideline on clinical nutrition in surgery - Update 2025 | Weimann, A et al.(2025,Clin Nutr) | The research content does not match. |
| 12 | Exercise And Physical Activity After Stoma SurgeryBest Practice Recommendations | Russell S et al.(2025,) | The research content does not match. |
| 13 | EAU Guidelines on Non-muscle-invasive Bladder Cancer (TaT1 and CIS) | P. Gontero Chair et al.(2025,) | The research content does not match. |
| 14 | NCCN Clinical Practice Guidelines in Oncology (NCCN Guidelines®)：Bladder Cancer Version 3.2025 | Thomas W. Flaig, M et al.(2025,NCCN) | The research content does not match. |
| 15 | NCCN Clinical Practice Guidelines in Oncology (NCCN Guidelines®):bladder cancer for patients | Philippe E. Spiess, M et al.(2025,NCCN) | The research content does not match. |
| 16 | 2025 Canadian Urological Association Guideline:Muscle-invasive bladder cancer | Kulkarni, G. S et al.(2025,) | The research content does not match. |
| 17 | Expert Consensus on Comprehensive Nutritional Management for Patients with Colorectal Cancer and Ostomy (2025 Edition) | Chinese Anti-Cancer Association, Committee on Tumor Nutrition. (2025, Chinese Journal of Gastrointestinal Surgery) | The research content does not match. |
| 18 | Summary of Best Evidence for Nutritional Management in Patients with Colorectal Cancer and Ostomy | Zhu Li et al. (2025, Chinese Journal of Modern Nursing) | The research content does not match. |
| 19 | Lifestyle Advice to Patients with Bladder Cancer: A National Survey of Dutch Urologists | Beeren, I et al.(2024,Bladder Cancer) | The research content does not match. |
| 20 | Outcome of perioperative immune enhancing nutrition in patients undergoing radical cystectomy | Sultan, S. M et al.(2024,African Journal of Urology) | The research content does not match. |
| 21 | Prehabilitation in patients undergoing bladder cancer surgery - A systematic review and meta-analysis | Steffens, D et al.(2024,The Canadian journal of urology) | The research content does not match. |
| 22 | Effects of immune-enhancing nutrition on radical cystectomy outcomes: Primary results from the randomized phase III double-blind clinical trial (S1600) | Hamilton-Reeves, J et al.(2024,Journal of Clinical Oncology) | The research content does not match. |
| 23 | Outcomes of a Dietary Intervention to Reduce Bladder Cancer Recurrence and Progression in Survivors of Non–Muscle-Invasive Bladder Cancer | Kim Yeary, K. H et al.(2024,NCCN Journal of the National Comprehensive Cancer Network) | The research content does not match. |
| 24 | Long term outcomes and impact on renal function following radical cystectomy | Tilala, Y. M et al.(2024,UROLOGIA JOURNAL) | The research content does not match. |
| 25 | Association of Pro-Inflammatory Diet, Smoking, and Alcohol Consumption with Bladder Cancer: Evidence from Case–Control and NHANES Studies from 1999 to 2020 | Teng, C.; Lu, W et al.(2024,Nutrients) | The research content does not match. |
| 26 | New Ostomy Patient Guide | (2024,United Ostomy Associations Of America) | The research content does not match. |
| 27 | Nutritional Therapy Guidelines for Radiation Therapy Patients (2024 Edition) | Li Tao et al. (2024, Electronic Journal of Cancer Metabolism and Nutrition) | The research content does not match. |
| 28 | European Association of Urology Guidelines on Muscle-invasive and Metastatic Bladder Cancer: Summary of the 2023 Guidelines | Alfred, W. J et al.(2024,Eur Urol) | The research content does not match. |
| 29 | French AFU Cancer Committee Guidelines - Update 2024-2026: Muscle-invasive bladder cancer (MIBC) | Roumiguié, M et al.(2024,Fr J Urol,) | The research content does not match. |
| 30 | Reporting perioperative complications of radical cystectomy: the influence of using standard methodology based on ICARUS and EAU quality criteria | Cinar, N. B et al.(2023,World Journal of Surgical Oncology) | The research content does not match. |
| 31 | Prognostic Ability of Nutritional Indices for Outcomes of Bladder Cancer: A Systematic Review and Meta-Analysis | Jiao, H et al.(2023,Urologia Internationalis) | The research content does not match. |
| 32 | Preoperative sarcopenia and malnutrition in patients scheduled to radical cystectomy. incidence and relationship to length of stay and postoperative complications after a prehabilitation program | Corcoy, M et al.(2023,Clinical Nutrition ESPEN) | The research content does not match. |
| 33 | Perioperative comparison of robot-assisted radical cystectomy and laparoscopic radical cystectomy: An update meta-analysis | Li, P et al.(2023,Asian journal of surgery) | The research content does not match. |
| 34 | Prognostic impact of geriatric nutritional risk index on patients with urological cancers: A meta-analysis | Wu, Q et al.(2023,Frontiers in Oncology) | The research content does not match. |
| 35 | Meat Intake and the Risk of Bladder Cancer: A Systematic Review and Meta-Analysis of Observational Studies | Yu, J. et al.(2023,Nutrition and Cancer) | The research content does not match. |
| 36 | Food groups and urologic cancers risk: a systematic review and meta-analysis of prospective studies | Qi, J et al.(2023,FRONTIERS IN NUTRITION) | The research content does not match. |
| 37 | Adherence to lifestyle recommendations after non-muscle invasive bladder cancer diagnosis and risk of recurrence | van Zutphen, M et al.(2023,AMERICAN JOURNAL OF CLINICAL NUTRITION,) | The research content does not match. |
| 38 | Longitudinal associations of adherence to lifestyle recommendations and health-related quality of life in patients with non-muscle invasive bladder cancer | Vidra, N et al.(2023,NTERNATIONAL JOURNAL OF CANCER) | The research content does not match. |
| 39 | The impact of early postoperative nutritional support on prognosis indicators in patients undergoing radical cystectomy | Zhang Chi et al. (2023, Journal of Nurse Training) | The research content does not match. |
| 40 | Application of Enhanced Recovery After Surgery (ERAS) Protocol in the Perioperative Management of Radical Cystectomy and Urinary Diversion | Fu Siming et al. (2023, Chongqing Medicine) | The research content does not match. |
| 41 | One-Year Follow-Up after Multimodal Prehabilitation Interventions in Radical Cystectomy | Thoft Jensen, B et al.(2023,Cancers) | The research content does not match. |
| 42 | LIVING WITH A UROSTOMY | (2023,United Ostomy Associations Of America) | The research content does not match. |
| 43 | [ESPEN practical guideline: Home enteral nutrition] | Bischoff, S. C et al.(2023,Nutr Hosp) | The research content does not match. |
| 44 | Expert Consensus on Nutritional Management for Cancer Patients in the Recovery Phase (2023 Edition) | Li Rongrong et al. (2023, Chinese Journal of Clinical Nutrition) | The research content does not match. |
| 45 | EffectiveNess of a multimodal preHAbilitation program in patieNts with bladder canCEr undergoing radical cystectomy: protocol of the ENHANCE multicentre randomised controlled trial | Akdemir, E et al.(2023,BMJ Open) | The research content does not match. |
| 46 | American Cancer Society nutrition and physical activity guideline for cancer survivors | Rock, C. L et al.(2022,CA Cancer Journal for Clinicians) | The research content does not match. |
| 47 | Dietary patterns and risk of bladder cancer: a systematic review and meta-analysis | Dianatinasab, M et al.(2022,BMC public health) | The research content does not match. |
| 48 | Consumption of Fruits, Vegetables and Bladder Cancer Risk: A Systematic Review and Meta-Analysis of Prospective Cohort Studies | Xenou, D et al.(2022,Nutrition and Cancer) | The research content does not match. |
| 49 | Dietary consumption of cruciferous vegetables and bladder cancer risk: A systematic review and meta-analysis | Yu, P. K et al.(2022,FRONTIERS IN NUTRITION) | The research content does not match. |
| 50 | Folate Intake and Risk of Urothelial Carcinoma: A Systematic Review and Meta-Analysis of Epidemiological Studies | Gu, Y et al.(202,NUTRITION AND CANCER-AN INTERNATIONAL JOURNAL) | The research content does not match. |
| 51 | STRONG for Surgery & Strong for Life — against all odds: intensive prehabilitation including smoking, nutrition, alcohol and physical activity for risk reduction in cancer surgery — a protocol for an RCT with nested interview study (STRONG-Cancer) | Tønnesen, H et al.(2022,Trials) | The research content does not match. |
| 52 | Epidemiology, Screening, and Prevention of Bladder Cancer | Lobo, N et al.(2022,Eur Urol Oncol) | The research content does not match. |
| 53 | Evidence Summary on Comprehensive Nursing Management for Adult Ostomy Patients | Wang Xia et al. (2022, Chinese Journal of Modern Nursing) | The research content does not match. |
| 54 | EffectiveNess of a multimodal preHAbilitation program in patieNts with bladder canCEr undergoing cystectomy. The ENHANCE randomized controlled trial | NL-OMON et al.(2022,BMJ Open) | The research content does not match. |
| 55 | Effects of early nutritional support treatment before hospitalization on clinical outcomes in patients with solid organ tumors | Hu, C et al.(2021,Clinical Nutrition ESPEN) | The research content does not match. |
| 56 | Enhanced Recovery After Surgery (ERAS®) after radical cystectomy—current data | Schulz, G. B et al.(2021,Urologe) | The research content does not match. |
| 57 | Multimodal Prehabilitation to Enhance Functional Capacity Following Radical Cystectomy: A Randomized Controlled Trial | Minnella, E. M et al.(2021,European Urology Focus) | The research content does not match. |
| 58 | Healthy behaviors are associated with positive outcomes for cancer survivors with ostomies: a cross-sectional study | Mo, J et al.(2021,JOURNAL OF CANCER SURVIVORSHIP) | The research content does not match. |
| 59 | Association Between Alcohol Consumption and Risk of Bladder Cancer: A Dose-Response Meta-Analysis of Prospective Cohort Studies | Lao, Y. F et al.(2021,FRONTIERS IN ONCOLOGY) | The research content does not match. |
| 60 | Nutritional Intervention and Health-Related Quality of Life in Patients Undergoing Radical Cystectomy -- A Randomized Controlled Trial. | Kort, A et al.(2021,Urologic Nursing) | The research content does not match. |
| 61 | Optimization of Enteral-Parenteral Sequential Nutritional Therapy in the Perioperative Period Following Radical Cystectomy with Ileal Conduit | Bai Qian et al. (2021, Chongqing Medicine) | The research content does not match. |
| 62 | Application of Enhanced Recovery After Surgery in Laparoscopic Radical Cystectomy with Ileal Conduit under Modular Surgical Workflow | Cao Zhi et al. (2021, International Journal of Surgery) | The research content does not match. |
| 63 | ESPEN practical guideline: Clinical nutrition in surgery | Weimann, A et al.(2021,Clin Nutr,) | The research content does not match. |
| 64 | ESPEN guideline on hospital nutrition | Thibault, R et al.(2021,Clin Nutr) | The research content does not match. |
| 65 | Canadian Urological Association guideline on the management of non-muscle-invasive bladder cancer - Full-text | Bhindi, B et al.(2021,Can Urol Assoc J,) | The research content does not match. |
| 66 | Systematic Review and Meta-Analysis of the Efficacy and Safety of Enhanced Recovery After Surgery vs. Conventional Recovery After Surgery on Perioperative Outcomes of Radical Cystectomy | Zhang, D et al.(2020,Frontiers in Oncology) | The research content does not match. |
| 67 | Nutrition risk and assessment process in patients with bladder cancer undergoing radical cystectomy | Michel, C et al.(2020,Urologic Oncology) | The research content does not match. |
| 68 | Randomised clinical trial of a gastrointestinal care bundle to reduce symptoms in patients with pelvic cancer undergoing chemoradiotherapy | White, K. L et al.(2020,BMJ Open Gastroenterology) | The research content does not match. |
| 69 | Modifiable lifestyle behaviours impact the health-related quality of life of bladder cancer survivors | Chung, J et al.(2020,BJU International) | The research content does not match. |
| 70 | Carotenoid Intake and Circulating Carotenoids Are Inversely Associated with the Risk of Bladder Cancer: A Dose-Response Meta-analysis | Wu, S et al.(2020,Advances in nutrition (Bethesda, Md.),) | The research content does not match. |
| 71 | Implementation of enhanced recovery after surgery in patients undergoing radical cystectomy: A retrospective cohort study | Zhang, H et al.(2020,EJSO) | The research content does not match. |
| 72 | Preliminary Application of Enhanced Recovery After Surgery Concept in Robot-Assisted Totally Intracorporeal STAPLER Radical Cystectomy | Wu Qi et al. (2020, Chinese Journal of Urology) | The research content does not match. |
| 73 | Perioperative nutrition: Recommendations from the ESPEN expert group | Lobo, D. N et al.(2020,Clin Nutr,) | The research content does not match. |
| 74 | A Proactive Approach to Bladder and Bowel Management in Adults | Doris Grinspun, R et al.(2020,FRONTIERS IN ONCOLOGY) | The research content does not match. |
| 75 | Continuing towards optimization of bladder cancer care in Canada: Summary of the third Bladder Cancer Canada-Canadian Urological Association-Canadian Urologic Oncology Group (BCC-CUA-CUOG) bladder cancer quality of care consensus meeting | Kassouf, W et al.(2020,Can Urol Assoc J) | The research content does not match. |
| 76 | Evidence Summary on the Implementation and Management of Oral Nutritional Supplementation in Patients with Malignant Tumors | Zhu Yunxia et al. (2020, Chinese Journal of Modern Nursing) | The research content does not match. |
| 77 | Guidelines for Tumor Immunonutrition Therapy | Cui Jiwei et al. (2020, Electronic Journal of Cancer Metabolism and Nutrition) | The research content does not match. |
| 78 | Continuing towards optimization of bladder cancer care in Canada: Summary of the third Bladder Cancer Canada-Canadian Urological Association-Canadian Urologic Oncology Group (BCC-CUA-CUOG) bladder cancer quality of care consensus meeting | Kassouf, W et al.(2020,Can Urol Assoc J) | The research content does not match. |
| 79 | Milk and Dairy Product Consumption and Bladder Cancer Risk: A Systematic Review and Meta-Analysis of Observational Studies | Bermejo, L. M et al.(2019,Advances in nutrition ) | The research content does not match. |
| 80 | Total parenteral nutrition versus early enteral nutrition after cystectomy: a meta-analysis of postoperative outcomes | Zeng, S et al.(2019,International Urology and Nephrology) | The research content does not match. |
| 81 | Usage of intravenous lidocaine infusion with enhanced recovery pathway in patients scheduled for open radical cystectomy: A randomized trial | Moeen, S. M et al.(2019,Pain Physician) | The research content does not match. |
| 82 | Low pretreatment prognostic nutritional index predicts poor survival of urologic cancer: An observational meta-analysis | Zhao, J. W et al.(2019,International Journal of Clinical and Experimental Medicine) | The research content does not match. |
| 83 | Association between coffee consumption and risk of bladder cancer in a meta-analysis of 16 prospective studies | Dai, Z. W et al.(2019,Nutrition and Metabolism) | The research content does not match. |
| 84 | Perioperative management of radical cystectomy in the Nordic countries | Oedorf, K et al.(2019,Scandinavian Journal of Urology) | The research content does not match. |
| 85 | Effectiveness of individualized nutritional interventions involving nurses in cancer patients | Li Yiqiong et al. (2019, Chinese Journal of Modern Nursing) | The research content does not match. |
| 86 | Pretreatment assessment and perioperative nutritional interventions for patients with bladder cancer undergoing radical cystectomy | Miyake, M et al.(2018,Translational Andrology and Urology) | The research content does not match. |
| 87 | Perioperative Immunonutrition Modulates Inflammatory Response after Radical Cystectomy: Results of a Pilot Randomized Controlled Clinical Trial | Hamilton-Reeves, J. M et al.(2018,Journal of Urology) | The research content does not match. |
| 88 | Red and processed meat consumption and risk of bladder cancer: a dose–response meta-analysis of epidemiological studies | Crippa, A et al.(2018,European Journal of Nutrition) | The research content does not match. |
| 89 | Prospective Implementation of Enhanced Recovery After Surgery Protocols to Radical Cystectomy | Pang, K. H et al.(2018,European Urology) | The research content does not match. |
| 90 | A randomized phase III double-blind clinical trial (S1600) evaluating the effect of immune-enhancing nutrition on radical cystectomy outcomes | Hamilton-Reeves, J et al.(2018,ournal of Clinical Oncology) | The research content does not match. |
| 91 | Body Mass Index, Diet-Related Factors, and Bladder Cancer Prognosis: A Systematic Review and Meta-Analysis | Westhoff, E et al.(2018,Bladder Cancer) | The research content does not match. |
| 92 | Enhanced recovery after surgery for radical cystectomy with ileal urinary diversion: a multi-institutional, randomized, controlled trial from the Chinese bladder cancer consortium | Lin, T et al.(2018,World journal of urology) | The research content does not match. |
| 93 | Nutritional implications for quality of life in bladder cancer survivors | Rosser, C. J et al.(2018,TRANSLATIONAL ANDROLOGY AND UROLOGY) | The research content does not match. |
| 94 | Dietary patterns and health-related quality of life in bladder cancer survivors. | Gopalakrishna, A et al.(2018,Urologic Oncology) | The research content does not match. |
| 95 | American Society for Enhanced Recovery and Perioperative Quality Initiative Joint Consensus Statement on Nutrition Screening and Therapy Within a Surgical Enhanced Recovery Pathway | Wischmeyer, P. E et al.(2018,Anesth Analg) | The research content does not match. |
| 96 | Improving patient journey and quality of care: Summary from the second Bladder Cancer Canada-Canadian Urological Association-Canadian Urologic Oncology Group (BCC-CUA-CUOG) bladder cancer quality of care consensus meeting | Kassouf, W et al.(2018,Can Urol Assoc J) | The research content does not match. |
| 97 | Interventions to reduce acute and late adverse gastrointestinal effects of pelvic radiotherapy for primary pelvic cancers | Lawrie, T. A et al.(2018,Cochrane Database of Systematic Reviews) | The research content does not match. |
| 98 | A gastrointestinal care bundle versus standard care to reduce the gastrointestinal effects of pelvic chemoradiotherapy: A pilot feasibility randomised controlled trial | White, K. L et al.(2017,Gut) | The research content does not match. |
| 99 | Effects of oral immunonutrition on cytokine profiles in bladder cancer patients: A pilot randomized controlled clinical trial | Stanley, A. et al.(2017,Journal of Parenteral and Enteral Nutrition) | The research content does not match. |
| 100 | Impact of an enhanced recovery after surgery programme in radical cystectomy. A cohort-comparative study | Casans-Francés, R et al.(2017,REVISTA ESPANOLA DE ANESTESIOLOGIA Y REANIMACION) | The research content does not match. |
| 101 | Surveillance report 2017 – Nutrition support for adults: oral nutrition support, enteral tube feeding and parenteral nutrition (2006) NICE guideline CG32 | (2017,NICE) | The research content does not match. |
| 102 | Oncological and functional outcomes of postoperative total parenteral nutrition after radical cystectomy in bladder cancer patients: A single-center randomized trial | Vidal, A et al.(2016,International Journal of Urology) | The research content does not match. |
| 103 | Enhanced Recovery After Robot-assisted Radical Cystectomy: EAU Robotic Urology Section Scientific Working Group Consensus View | Collins, J. W et al.(2016,European Urology) | The research content does not match. |
| 104 | A Prospective Randomized Trial of the Effects of Early Enteral Feeding After Radical Cystectomy | Deibert, C. M et al.(2016,Urology) | The research content does not match. |
| 105 | Modifiable risk factors for the prevention of bladder cancer: a systematic review of meta-analyses | Al-Zalabani, A. H et al.(2016,European Journal of Epidemiology) | The research content does not match. |
| 106 | A pilot study of high-arginine nutritional supplementation prior to radical cystectomy | Lyon, T et al.(2016,Canadian Urological Association Journal) | The research content does not match. |
| 107 | Effects of Immunonutrition for Cystectomy on Immune Response and Infection Rates: A Pilot Randomized Controlled Clinical Trial | Hamilton-Reeves, J. M et al.(2016,European Urology) | The research content does not match. |
| 108 | Increasing vegetable intakes: rationale and systematic review of published interventions | Appleton, K. M et al.(2016,EUROPEAN JOURNAL OF NUTRITION) | The research content does not match. |
| 109 | Guideline on Muscle-Invasive and Metastatic Bladder Cancer (European Association of Urology guideline): American Society of Clinical Oncology Clinical Practice Guideline Endorsement | (2016,ASCO, U. S) | The research content does not match. |
| 110 | Nutritional Therapy Guidelines for Chemotherapy Patients | Chinese Anti-Cancer Association et al. (2016, Electronic Journal of Tumor Metabolism and Nutrition) | The research content does not match. |
| 111 | Physical Prehabilitation is Feasible and Effective in Patients With Advanced Bladder Cancer | Jensen, B. T et al.(2015,Cancer Nursing) | The research content does not match. |
| 112 | Reduced length of stay in radical cystectomy patients with oral versus parenteral post-operative nutrition protocol | Declercq, P et al.(2015,INTERNATIONAL JOURNAL OF CLINICAL PHARMACY) | The research content does not match. |
| 113 | Nutritional support at the end of life | Orrevall, Y et al.(2015,Nutrition) | The research content does not match. |
| 114 | Care of dying adults in the last days of life (NG31) | et al.(2015,FRONTIERS IN ONCOLOGY) | The research content does not match. |
| 115 | Discharge planning for a patient with a new ostomy: best practice for clinicians | Prinz, A. et al.(2015,J Wound Ostomy Continence Nurs) | The research content does not match. |
| 116 | The Application of Fast-track Recovery in Radical Cystectomy with Ileal Urinay Diversion: a Multi-institutional Randomized Controlled Trial | ChiCTR-ICR et al.(2025,FRONTIERS IN ONCOLOGY) | The research content does not match. |
| 117 | Is it necessary to insert nasogastric tube routinely after radical cystectomy with urinary diversion? A meta-analysis | Zhao, T et al.(2014,International Journal of Clinical and Experimental Medicine) | The research content does not match. |
| 118 | Flavonoid and lignan intake in relation to bladder cancer risk in the European Prospective Investigation into Cancer and Nutrition (EPIC) study | Zamora-Ros, R et al.(2014,BRITISH JOURNAL OF CANCER) | The research content does not match. |
| 119 | Guidelines for perioperative care after radical cystectomy for bladder cancer: Enhanced recovery after surgery (ERAS®) society recommendations | Cerantola, Y et al.(2013,Clinical Nutrition) | The research content does not match. |
| 120 | A prospective randomized multicenter study of Turkish Society of Urooncology comparing two different mechanical bowel preparation methods for radical cystectomy | Aslan, G et al.(2013,Urologic Oncology) | The research content does not match. |
| 121 | Early removal of nasogastric tube is beneficial for patients undergoing radical cystectomy with urinary diversion | Adamakis, I et al.(2011,International Braz J Urol,) | The research content does not match. |
| 122 | Early removal of nasogastric tube is beneficial for patients undergoing radical cystectomy with urinary diversion | Adamakis, I et al.(2011,International Braz J Urol) | The research content does not match. |
| 123 | A.S.P.E.N. Clinical guidelines: Nutrition support therapy during adult anticancer treatment and in hematopoietic cell transplantation | August, D. A et al.(2009,Journal of Parenteral and Enteral Nutrition) | The research content does not match. |
| 124 | CONTINENT UROSTOMY GUIDE | Ilene Fleischer, M et al.(2009,UOAA) | The research content does not match. |
| 125 | Current perioperative management of radical cystectomy with intestinal urinary reconstruction for muscle-invasive bladder cancer and reduction of the incidence of postoperative ileus | Maffezzini, M et al.(2008,SURGICAL ONCOLOGY-OXFORD) | The research content does not match. |
| 126 | Palliative Care in Adolescents and Young Adults With Cancer | Emily E Johnston et al.(2024,Journal of Clinical Oncology) | The research content does not match. |
| 127 | Improving Outcomes Guidance on Cancer Services：Improving Outcomes in Urological Cancers | (2002,NICE) | The research content does not match. |
| 128 | Ina, K.I.A.O. and D.A.T.O. Early Detection, Früherkennung, Diagnose, Therapie und Nachsorge des Harnblasenkarzinoms. S3-LL (Leitlinienprogramm Onkologie von AWMF, DKG und DKH). | (2025,AWMF) | Non-Chinese and non-English literature |
| 129 | 2024 CUA-GUMOC Expert Report: Management of unresectable locally advanced and metastatic urothelial carcinoma | Carlos E Stecca et al.(2024,Cuaj-canadian Urological Association Journal) | Review, Conference Abstract |
| 130 | Comprehensive care for patients with bladder cancer: Addressing the interplay of physical, nutritional, and emotional well-being | Bergerot, C. D et al.(2025,Cancer) | Review, Conference Abstract |
| 131 | Advances in diagnosis and treatment of bladder cancer | Lopez-Beltran, A et al.(2024,BMJ) | Review, Conference Abstract |
| 132 | Bladder cancer | Lars Dyrskjøt et al.(2024,Nat Rev Dis Primers) | Review, Conference Abstract |
| 133 | Radical Cystectomy Perioperative Care Redesign | Matulewicz, R. S et al.(2015,Urology) | Review, Conference Abstract |
| 134 | Perioperative Strategies to Reduce Postoperative Complications After Radical Cystectomy | Tomaszewski, J et al.(2015,Current Urology Reports) | Review, Conference Abstract |
| 135 | Nutritional interventions for reducing gastrointestinal toxicity in adults undergoing radical pelvic radiotherapy | Henson, C. C et al.(2013,Cochrane Database of Systematic Reviews) | Review, Conference Abstract |

**Appendix C**

**Table S1.** Value Assignment for Expert Judgment Bases

| Judgment Basis | Degree of Influence on Expert Judgment | | |
| --- | --- | --- | --- |
|  | High(1.0) | Medium(0.8) | Low(0.5) |
| Work Experience Judgment | 0.5 | 0.4 | 0. |
| Theoretical Knowledge Analysis | 0.3 | 0.2 | 0.1 |
| Reference to Domestic and International Literature | 0.1 | 0.1 | 0.05 |
| Reference to Domestic and International Literature | 0.1 | 0.1 | 0.05 |

**Table S2.** Expert Authority Level (n=17)

| Expert | Judgment criteria | | | | Coefficient of determination  (Ca) | Familiarity coefficient  (Cs) | Authoritative coefficient  (Cr) |
| --- | --- | --- | --- | --- | --- | --- | --- |
|  | Work Experience | Theoretical Analysis | Domestic and international related literature | Personal intuition |  |  |  |
| 1 | 0.5 | 0.3 | 0.1 | 0.05 | 0.95 | 1 | 0.88 |
| 2 | 0.3 | 0.3 | 0.1 | 0.05 | 0.75 | 0.6 |  |
| 3 | 0.5 | 0.3 | 0.1 | 0.1 | 1 | 1 |  |
| 4 | 0.5 | 0.3 | 0.05 | 0.1 | 0.95 | 1 |  |
| 5 | 0.5 | 0.3 | 0.1 | 0.05 | 0.95 | 1 |  |
| 6 | 0.4 | 0.3 | 0.1 | 0.05 | 0.85 | 0.8 |  |
| 7 | 0.5 | 0.3 | 0.05 | 0.1 | 0.95 | 0.6 |  |
| 8 | 0.4 | 0.3 | 0.1 | 0.1 | 0.9 | 1 |  |
| 9 | 0.5 | 0.3 | 0.1 | 0.05 | 0.95 | 1 |  |
| 10 | 0.4 | 0.3 | 0.1 | 0.05 | 0.85 | 0.8 |  |
| 11 | 0.5 | 0.2 | 0.1 | 0.05 | 0.85 | 1 |  |
| 12 | 0.4 | 0.3 | 0.1 | 0.05 | 0.85 | 1 |  |
| 13 | 0.5 | 0.3 | 0.05 | 0.1 | 0.95 | 1 |  |
| 14 | 0.4 | 0.3 | 0.1 | 0.1 | 0.9 | 0.4 |  |
| 15 | 0.5 | 0.2 | 0.1 | 0.1 | 0.9 | 0.6 |  |
| 16 | 0.4 | 0.3 | 0.1 | 0.1 | 0.9 | 0.6 |  |
| 17 | 0.5 | 0.3 | 0.1 | 0.05 | 0.95 | 1 |  |
| Total |  | | | | 15.4 | 14.4 |  |

**Table S3.** Expert Opinion Consistency Table

|  | Coordination coefficient(W) | 2 | P value |
| --- | --- | --- | --- |
| 1 round(n=17) | 0.250 | 441.420 | ＜0.001 |
| 2 round(n=15) | 0.313 | 473.497 | ＜0.001 |

**Table S4.** Summary of Expert Opinions and Revision Table

|  | Entry | Reason |
| --- | --- | --- |
| Delete | Avoid eating vegetables within 24 hours before surgery (original clause 30) | Low level of evidence and high controversy |
|  | Establish a three-tier nutritional diagnosis system comprising nutritional screening, nutritional assessment, and comprehensive evaluation. (Original Clause 4) | Proceed to the discussion section of the subsequent article. |
|  | Early recommendation of oral analgesics, with epidural analgesia not routinely used (original item 35) | Content with low relevance to nutritional information |
|  | Remove the painkiller Alvimopan, an analgesic (formerly Article 67) | Currently not widely available for sale in China or included in medical insurance, and with a relatively high price, it is not suitable for clinical promotion. |
| Merge similar entries | (② Adding medium- and long-chain triglyceride (MCT) emulsions to parenteral nutrition (PN) can improve lipid metabolism and reduce immunosuppressive responses; structured lipid emulsions promote balanced metabolism and protect liver function; fish oil emulsions help regulate inflammatory responses and improve organ function; olive oil emulsions reduce lipid peroxidation; multiple-oil emulsions (SMOF), which optimize fatty acid composition, offer clinical benefits and contain 30% soybean oil, 30% coconut oil, 25% olive oil, 15% fish oil, and vitamin E [1]. ③ Adding glutamine (Gln) dipeptides to PN helps maintain intestinal mucosal barrier function and immune function, reducing infectious complications; however, Gln dipeptide supplementation is not recommended in critically ill patients with severe hepatic or renal dysfunction or shock [1].) (Original items 69 simplify and combined into item 73) | The content is redundant. |
|  | Deletion of entries: Traditional Chinese medicine acupoint stimulation techniques such as electroacupuncture, TEA, manual acupuncture, ear pressure, finger pressure, or combinations thereof (62); and integration of other TCM-related content (unified under the "Auxiliary Interventions" module) (originally 61.67.68) into (81.82.83). | Entry 62: Both importance and feasibility scores are below 4.0, with poor expert consensus.  The evidence base for traditional Chinese medicine is weak and inconsistent with mainstream guidelines; it is only considered an adjunctive intervention method. |
| Suggestion | Nutritional screening phase: MNA-SF is used to screen for malnutrition in elderly patients. | After discussion, NRS 2002 was maintained as the preferred tool due to its ability to simultaneously assess disease and nutritional status, strong evidence base, extensive global validation, high sensitivity and specificity for cancer patients, and recommendations from multiple clinical guidelines. |
| Suggestion | Nutritional Comprehensive Assessment Phase: Laboratory tests such as TNF-α, IL-1, and IBI are difficult to perform in primary healthcare facilities due to limited equipment and high costs. Given the basic nature of testing capabilities at these facilities, it is recommended to reduce the number of related laboratory indicators. The four-level evaluation is considered an "ideal recommendation" for higher-level medical centers or research settings; in routine clinical practice, a simplified approach using CRP, albumin, prealbumin, and clinical NIS symptom scoring can be adopted, with a note indicating that "these tests may be selectively performed based on institutional resources." | After group discussion, the expert's advice was accepted. |

**Appendix D**

**Table S1.** Importance scores of the items in the first round

| Item | Mean | Standard deviation (SD) | Coefficient of variation (CV) |
| --- | --- | --- | --- |
| A1 | 4.65 | 0.93 | 0.2 |
| A2 | 4.71 | 0.77 | 0.16 |
| A3 | 4.65 | 0.79 | 0.17 |
| A4 | 4.35 | 0.86 | 0.2 |
| A5 | 4.65 | 0.7 | 0.15 |
| A6 | 4.29 | 0.99 | 0.23 |
| A7 | 4.18 | 0.95 | 0.23 |
| A8 | 4.47 | 0.8 | 0.18 |
| A9 | 4.53 | 0.87 | 0.19 |
| A10 | 4.71 | 0.77 | 0.16 |
| A11 | 4.18 | 1.24 | 0.3 |
| A12 | 4.65 | 0.93 | 0.2 |
| A13 | 4.65 | 0.7 | 0.15 |
| A14 | 4.59 | 0.87 | 0.19 |
| A15 | 4.53 | 0.94 | 0.21 |
| A16 | 4.41 | 0.94 | 0.21 |
| A17 | 4.24 | 1.25 | 0.3 |
| A18 | 4.71 | 0.47 | 0.1 |
| A19 | 4.71 | 0.59 | 0.12 |
| A20 | 4.29 | 1.05 | 0.24 |
| A21 | 4.41 | 0.87 | 0.2 |
| A22 | 4.65 | 0.61 | 0.13 |
| A23 | 4.41 | 0.94 | 0.21 |
| A24 | 4.65 | 0.7 | 0.15 |
| A25 | 4.53 | 0.87 | 0.19 |
| A26 | 4.53 | 0.8 | 0.18 |
| A27 | 3.35 | 1.37 | 0.41 |
| A28 | 3.76 | 1.03 | 0.27 |
| A29 | 4.29 | 1.26 | 0.29 |
| A30 | 2.65 | 1.66 | 0.63 |
| A31 | 4.06 | 1.3 | 0.32 |
| A32 | 4.41 | 1.28 | 0.29 |
| A33 | 3.76 | 1.56 | 0.41 |
| A34 | 3.94 | 1.09 | 0.28 |
| A35 | 2.65 | 1.37 | 0.52 |
| A36 | 4.18 | 1.13 | 0.27 |
| A37 | 3.41 | 1.33 | 0.39 |
| A38 | 3.71 | 0.99 | 0.27 |
| A39 | 3.12 | 1.41 | 0.45 |
| A40 | 4.47 | 0.72 | 0.16 |
| A41 | 4.41 | 0.94 | 0.21 |
| A42 | 4.76 | 0.56 | 0.12 |
| A43 | 4.47 | 1.07 | 0.24 |
| A44 | 4.71 | 0.59 | 0.12 |
| A45 | 3.29 | 1.53 | 0.46 |
| A46 | 4.65 | 0.7 | 0.15 |
| A47 | 4.41 | 0.94 | 0.21 |
| A48 | 4.71 | 0.47 | 0.1 |
| A49 | 4.59 | 0.8 | 0.17 |
| A50 | 4.71 | 0.77 | 0.16 |
| A51 | 4.18 | 0.95 | 0.23 |
| A52 | 4.59 | 0.8 | 0.17 |
| A53 | 4.59 | 0.71 | 0.15 |
| A54 | 4.71 | 0.59 | 0.12 |
| A55 | 4.35 | 0.79 | 0.18 |
| A56 | 3.29 | 1.61 | 0.49 |
| A57 | 4.18 | 0.88 | 0.21 |
| A58 | 3.53 | 1.42 | 0.4 |
| A59 | 4.53 | 0.72 | 0.16 |
| A60 | 4.59 | 0.62 | 0.13 |
| A61 | 2.82 | 1.42 | 0.5 |
| A62 | 4.76 | 0.44 | 0.09 |
| A63 | 4.71 | 0.47 | 0.1 |
| A64 | 4.47 | 0.87 | 0.19 |
| A65 | 3.41 | 1.62 | 0.47 |
| A66 | 4.47 | 0.94 | 0.21 |
| A67 | 2.76 | 1.52 | 0.55 |
| A68 | 3.35 | 1.17 | 0.35 |
| A69 | 2.94 | 1.48 | 0.5 |
| A70 | 4.12 | 1.65 | 0.4 |
| A71 | 4.59 | 0.8 | 0.17 |
| A72 | 4.65 | 0.7 | 0.15 |
| A73 | 4.76 | 0.44 | 0.09 |
| A74 | 4.82 | 0.39 | 0.08 |
| A75 | 4.18 | 0.88 | 0.21 |
| A76 | 4.76 | 0.56 | 0.12 |
| A77 | 4.71 | 0.59 | 0.12 |
| A78 | 4.53 | 0.87 | 0.19 |
| A79 | 4.59 | 0.94 | 0.2 |
| A80 | 4.47 | 0.87 | 0.19 |
| A81 | 4.76 | 0.56 | 0.12 |
| A82 | 4.76 | 0.56 | 0.12 |
| A83 | 4.76 | 0.56 | 0.12 |
| A84 | 4.53 | 0.8 | 0.18 |
| A85 | 4.59 | 0.62 | 0.13 |
| A86 | 4.76 | 0.44 | 0.09 |
| A87 | 4.76 | 0.56 | 0.12 |
| A88 | 4.82 | 0.39 | 0.08 |
| A89 | 4.65 | 0.61 | 0.13 |
| A90 | 3.59 | 1.62 | 0.45 |
| A91 | 4.12 | 0.93 | 0.23 |
| A92 | 4.12 | 0.93 | 0.23 |
| A93 | 4.18 | 1.01 | 0.24 |
| A94 | 4.59 | 0.94 | 0.2 |
| A95 | 4.65 | 0.7 | 0.15 |
| A96 | 3.35 | 1.62 | 0.48 |
| A97 | 4.76 | 0.44 | 0.09 |
| A98 | 4.35 | 0.93 | 0.21 |
| A99 | 4.59 | 0.94 | 0.2 |
| A100 | 4.65 | 0.86 | 0.18 |
| A101 | 4.47 | 1.07 | 0.24 |
| A102 | 3.35 | 1.32 | 0.39 |
| A103 | 4.65 | 0.61 | 0.13 |
| A104 | 4.53 | 0.72 | 0.16 |
| A105 | 4.76 | 0.56 | 0.12 |

**Table S2.** Feasibility scores of the items in the first round

| Item | Mean | Standard deviation (SD) | CV(%) |
| --- | --- | --- | --- |
| A1 | 4.59 | 0.94 | 20.5 |
| A2 | 4.71 | 0.77 | 16.3 |
| A3 | 4.71 | 0.77 | 16.3 |
| A4 | 4.35 | 0.86 | 19.8 |
| A5 | 4.71 | 0.69 | 14.6 |
| A6 | 4.35 | 0.93 | 21.4 |
| A7 | 4.18 | 1.01 | 24.2 |
| A8 | 4.47 | 0.8 | 17.9 |
| A9 | 4.65 | 0.79 | 17 |
| A10 | 4.76 | 0.56 | 11.8 |
| A11 | 4.24 | 1.2 | 28.3 |
| A12 | 4.65 | 0.79 | 17 |
| A13 | 4.59 | 0.62 | 13.5 |
| A14 | 4.59 | 0.94 | 20.5 |
| A15 | 4.65 | 0.7 | 15.1 |
| A16 | 4.47 | 0.94 | 21 |
| A17 | 4.35 | 1.17 | 26.9 |
| A18 | 4.71 | 0.69 | 14.6 |
| A19 | 4.76 | 0.56 | 11.8 |
| A20 | 4.47 | 1.01 | 22.6 |
| A21 | 4.41 | 0.8 | 18.1 |
| A22 | 4.71 | 0.69 | 14.6 |
| A23 | 4.53 | 0.8 | 17.7 |
| A24 | 4.71 | 0.59 | 12.5 |
| A25 | 4.53 | 0.8 | 17.7 |
| A26 | 4.65 | 0.79 | 17 |
| A27 | 3.41 | 1.33 | 39 |
| A28 | 3.76 | 1.03 | 27.4 |
| A29 | 4.59 | 1 | 21.8 |
| A30 | 2.82 | 1.67 | 59.2 |
| A31 | 4.06 | 1.3 | 32 |
| A32 | 4.41 | 1.33 | 30.2 |
| A33 | 3.94 | 1.48 | 37.6 |
| A34 | 4.18 | 1.07 | 25.6 |
| A35 | 2.88 | 1.45 | 50.3 |
| A36 | 4.35 | 0.93 | 21.4 |
| A37 | 3.41 | 1.46 | 42.8 |
| A38 | 3.88 | 1.05 | 27.1 |
| A39 | 3.35 | 1.5 | 44.8 |
| A40 | 4.65 | 0.61 | 13.1 |
| A41 | 4.47 | 0.94 | 21 |
| A42 | 4.76 | 0.56 | 11.8 |
| A43 | 4.59 | 1 | 21.8 |
| A44 | 4.76 | 0.56 | 11.8 |
| A45 | 3.35 | 1.46 | 43.6 |
| A46 | 4.59 | 0.87 | 19 |
| A47 | 4.47 | 0.94 | 21 |
| A48 | 4.71 | 0.59 | 12.5 |
| A49 | 4.59 | 0.94 | 20.5 |
| A50 | 4.82 | 0.53 | 11 |
| A51 | 4.18 | 0.95 | 22.7 |
| A52 | 4.53 | 0.8 | 17.7 |
| A53 | 4.59 | 0.71 | 15.5 |
| A54 | 4.76 | 0.56 | 11.8 |
| A55 | 4.35 | 0.86 | 19.8 |
| A56 | 3.41 | 1.5 | 44 |
| A57 | 4.24 | 0.9 | 21.2 |
| A58 | 3.71 | 1.4 | 37.7 |
| A59 | 4.65 | 0.7 | 15.1 |
| A60 | 4.65 | 0.7 | 15.1 |
| A61 | 3 | 1.41 | 47 |
| A62 | 4.82 | 0.39 | 8.1 |
| A63 | 4.76 | 0.56 | 11.8 |
| A64 | 4.47 | 0.94 | 21 |
| A65 | 3.47 | 1.5 | 43.2 |
| A66 | 4.59 | 0.87 | 19 |
| A67 | 2.82 | 1.47 | 52.1 |
| A68 | 3.47 | 1.23 | 35.4 |
| A69 | 3 | 1.5 | 50 |
| A70 | 4.41 | 1 | 22.7 |
| A71 | 4.65 | 0.79 | 17 |
| A72 | 4.59 | 0.94 | 20.5 |
| A73 | 4.76 | 0.44 | 9.2 |
| A74 | 4.88 | 0.33 | 6.8 |
| A75 | 4.18 | 0.95 | 22.7 |
| A76 | 4.76 | 0.56 | 11.8 |
| A77 | 4.71 | 0.59 | 12.5 |
| A78 | 4.59 | 0.8 | 17.4 |
| A79 | 4.65 | 0.7 | 15.1 |
| A80 | 4.65 | 0.79 | 17 |
| A81 | 4.76 | 0.56 | 11.8 |
| A82 | 4.82 | 0.53 | 11 |
| A83 | 4.82 | 0.39 | 8.1 |
| A84 | 4.59 | 0.8 | 17.4 |
| A85 | 4.71 | 0.59 | 12.5 |
| A86 | 4.76 | 0.44 | 9.2 |
| A87 | 4.71 | 0.69 | 14.6 |
| A88 | 4.82 | 0.53 | 11 |
| A89 | 4.76 | 0.44 | 9.2 |
| A90 | 4 | 1.5 | 37.5 |
| A91 | 4.12 | 0.93 | 22.6 |
| A92 | 4.29 | 0.92 | 21.4 |
| A93 | 4.29 | 0.92 | 21.4 |
| A94 | 4.65 | 0.79 | 17 |
| A95 | 4.76 | 0.56 | 11.8 |
| A96 | 3.59 | 1.62 | 45.1 |
| A97 | 4.82 | 0.53 | 11 |
| A98 | 4.29 | 0.99 | 23.1 |
| A99 | 4.59 | 0.94 | 20.5 |
| A100 | 4.82 | 0.53 | 11 |
| A101 | 4.53 | 0.94 | 20.8 |
| A102 | 3.76 | 1.52 | 40.4 |
| A103 | 4.76 | 0.56 | 11.8 |
| A104 | 4.59 | 0.8 | 17.4 |
| A105 | 4.82 | 0.53 | 11 |

**Table S3.** Importance scores of the items in the second round

| Item | Mean | Standard deviation (SD) | Coefficient of variation (CV) |
| --- | --- | --- | --- |
| A1 | 4.93 | 0.26 | 0.05 |
| A2 | 4.93 | 0.26 | 0.05 |
| A3 | 4.87 | 0.52 | 0.11 |
| A4 | 4.8 | 0.41 | 0.09 |
| A5 | 4.47 | 0.83 | 0.19 |
| A6 | 4.4 | 0.83 | 0.19 |
| A7 | 4.93 | 0.26 | 0.05 |
| A8 | 4.8 | 0.41 | 0.09 |
| A9 | 4.93 | 0.26 | 0.05 |
| A10 | 4.87 | 0.35 | 0.07 |
| A11 | 4.93 | 0.26 | 0.05 |
| A12 | 4.93 | 0.26 | 0.05 |
| A13 | 4.73 | 0.59 | 0.13 |
| A14 | 4.8 | 0.41 | 0.09 |
| A15 | 4.87 | 0.35 | 0.07 |
| A16 | 4.93 | 0.26 | 0.05 |
| A17 | 4.87 | 0.35 | 0.07 |
| A18 | 4.53 | 0.83 | 0.18 |
| A19 | 4.73 | 0.59 | 0.13 |
| A20 | 4.8 | 0.41 | 0.09 |
| A21 | 4.8 | 0.41 | 0.09 |
| A22 | 4.87 | 0.35 | 0.07 |
| A23 | 4.93 | 0.26 | 0.05 |
| A24 | 4.93 | 0.26 | 0.05 |
| A25 | 3.73 | 1.44 | 0.39 |
| A26 | 3.87 | 0.99 | 0.26 |
| A27 | 4.27 | 1.28 | 0.3 |
| A28 | 4 | 1.46 | 0.37 |
| A29 | 4.73 | 0.88 | 0.19 |
| A30 | 3.6 | 1.68 | 0.47 |
| A31 | 4.53 | 1.06 | 0.23 |
| A32 | 4.4 | 0.83 | 0.19 |
| A33 | 3.87 | 0.92 | 0.24 |
| A34 | 3.33 | 1.4 | 0.42 |
| A35 | 4.6 | 0.63 | 0.14 |
| A36 | 4.93 | 0.26 | 0.05 |
| A37 | 4.73 | 0.7 | 0.15 |
| A38 | 4.8 | 0.41 | 0.09 |
| A39 | 4.93 | 0.26 | 0.05 |
| A40 | 4.87 | 0.35 | 0.07 |
| A41 | 3.33 | 1.54 | 0.46 |
| A42 | 4.73 | 0.46 | 0.1 |
| A43 | 4.53 | 0.83 | 0.18 |
| A44 | 4.8 | 0.41 | 0.09 |
| A45 | 4.6 | 0.83 | 0.18 |
| A46 | 4.93 | 0.26 | 0.05 |
| A47 | 4.13 | 0.83 | 0.2 |
| A48 | 4.53 | 0.64 | 0.14 |
| A49 | 4.53 | 0.74 | 0.16 |
| A50 | 4.87 | 0.35 | 0.07 |
| A51 | 4.53 | 0.74 | 0.16 |
| A52 | 3.33 | 1.5 | 0.45 |
| A53 | 4.13 | 0.83 | 0.2 |
| A54 | 3.73 | 1.58 | 0.42 |
| A55 | 4.8 | 0.41 | 0.09 |
| A56 | 4.53 | 0.64 | 0.14 |
| A57 | 4.8 | 0.41 | 0.09 |
| A58 | 4.73 | 0.46 | 0.1 |
| A59 | 4 | 1.46 | 0.37 |
| A60 | 3.93 | 1.33 | 0.34 |
| A61 | 4.6 | 0.83 | 0.18 |
| A62 | 3.4 | 1.64 | 0.48 |
| A63 | 4.93 | 0.26 | 0.05 |
| A64 | 4.93 | 0.26 | 0.05 |
| A65 | 4.67 | 0.62 | 0.13 |
| A66 | 4.73 | 0.46 | 0.1 |
| A67 | 4.67 | 0.49 | 0.1 |
| A68 | 4.73 | 0.59 | 0.13 |
| A69 | 4.8 | 0.41 | 0.09 |
| A70 | 4.13 | 0.83 | 0.2 |
| A71 | 4.8 | 0.56 | 0.12 |
| A72 | 4.73 | 0.46 | 0.1 |
| A73 | 4.53 | 0.74 | 0.16 |
| A74 | 4.73 | 0.46 | 0.1 |
| A75 | 4.93 | 0.26 | 0.05 |
| A76 | 4.8 | 0.41 | 0.09 |
| A77 | 4.93 | 0.26 | 0.05 |
| A78 | 4.73 | 0.59 | 0.13 |
| A79 | 4.73 | 0.7 | 0.15 |
| A80 | 4.87 | 0.35 | 0.07 |
| A81 | 2.87 | 1.51 | 0.53 |
| A82 | 2.87 | 1.41 | 0.49 |
| A83 | 3.13 | 1.06 | 0.34 |
| A84 | 4.93 | 0.26 | 0.05 |
| A85 | 4.87 | 0.35 | 0.07 |
| A86 | 4.67 | 0.49 | 0.1 |
| A87 | 3.47 | 1.55 | 0.45 |
| A88 | 3.8 | 0.86 | 0.23 |
| A89 | 4.27 | 0.8 | 0.19 |
| A90 | 3.8 | 1.15 | 0.3 |
| A91 | 4.8 | 0.41 | 0.09 |
| A92 | 4.67 | 0.9 | 0.19 |
| A93 | 3.6 | 1.76 | 0.49 |
| A94 | 4.93 | 0.26 | 0.05 |
| A95 | 4.13 | 0.83 | 0.2 |
| A96 | 4.73 | 0.46 | 0.1 |
| A97 | 4.67 | 0.62 | 0.13 |
| A98 | 4.4 | 1.12 | 0.25 |
| A99 | 3.33 | 1.4 | 0.42 |
| A100 | 4.87 | 0.35 | 0.07 |
| A101 | 4.6 | 0.83 | 0.18 |
| A102 | 4.8 | 0.56 | 0.12 |

**Table S4.** Feasibility scores of the items in the second round

| Item | Mean | Standard deviation (SD) | CV(%) |
| --- | --- | --- | --- |
| A1 | 4.93 | 0.26 | 5.2 |
| A2 | 4.93 | 0.26 | 5.2 |
| A3 | 4.87 | 0.35 | 7.2 |
| A4 | 4.87 | 0.35 | 7.2 |
| A5 | 4.53 | 0.74 | 16.4 |
| A6 | 4.6 | 0.74 | 16 |
| A7 | 4.93 | 0.26 | 5.2 |
| A8 | 4.87 | 0.35 | 7.2 |
| A9 | 4.93 | 0.26 | 5.2 |
| A10 | 4.87 | 0.35 | 7.2 |
| A11 | 4.93 | 0.26 | 5.2 |
| A12 | 4.93 | 0.26 | 5.2 |
| A13 | 4.8 | 0.56 | 11.7 |
| A14 | 4.87 | 0.35 | 7.2 |
| A15 | 4.93 | 0.26 | 5.2 |
| A16 | 5 | 0 | 0 |
| A17 | 4.93 | 0.26 | 5.2 |
| A18 | 4.67 | 0.82 | 17.5 |
| A19 | 4.8 | 0.56 | 11.7 |
| A20 | 4.8 | 0.56 | 11.7 |
| A21 | 4.8 | 0.56 | 11.7 |
| A22 | 4.87 | 0.35 | 7.2 |
| A23 | 4.93 | 0.26 | 5.2 |
| A24 | 4.93 | 0.26 | 5.2 |
| A25 | 4.2 | 1.21 | 28.8 |
| A26 | 4 | 1 | 25 |
| A27 | 4.53 | 0.92 | 20.3 |
| A28 | 4.33 | 1.29 | 29.8 |
| A29 | 4.8 | 0.77 | 16.1 |
| A30 | 3.8 | 1.57 | 41.3 |
| A31 | 4.6 | 0.91 | 19.8 |
| A32 | 4.53 | 0.74 | 16.4 |
| A33 | 4.13 | 0.92 | 22.2 |
| A34 | 3.93 | 1.16 | 29.5 |
| A35 | 4.67 | 0.82 | 17.5 |
| A36 | 5 | 0 | 0 |
| A37 | 4.73 | 0.7 | 14.8 |
| A38 | 4.87 | 0.35 | 7.2 |
| A39 | 4.93 | 0.26 | 5.2 |
| A40 | 4.87 | 0.35 | 7.2 |
| A41 | 3.53 | 1.41 | 39.9 |
| A42 | 4.8 | 0.41 | 8.6 |
| A43 | 4.73 | 0.7 | 14.8 |
| A44 | 4.87 | 0.35 | 7.2 |
| A45 | 4.73 | 0.7 | 14.8 |
| A46 | 4.93 | 0.26 | 5.2 |
| A47 | 4.4 | 0.83 | 18.9 |
| A48 | 4.6 | 0.74 | 16 |
| A49 | 4.6 | 0.74 | 16 |
| A50 | 4.93 | 0.26 | 5.2 |
| A51 | 4.67 | 0.72 | 15.4 |
| A52 | 3.6 | 1.35 | 37.5 |
| A53 | 4.47 | 0.92 | 20.5 |
| A54 | 4.47 | 0.92 | 20.5 |
| A55 | 4.8 | 0.56 | 11.7 |
| A56 | 4.4 | 1.12 | 25.5 |
| A57 | 4.8 | 0.56 | 11.7 |
| A58 | 4.8 | 0.56 | 11.7 |
| A59 | 4.27 | 1.03 | 24.1 |
| A60 | 4.47 | 1.06 | 23.7 |
| A61 | 4.67 | 0.82 | 17.5 |
| A62 | 3.33 | 1.59 | 47.7 |
| A63 | 4.93 | 0.26 | 5.2 |
| A64 | 5 | 0 | 0 |
| A65 | 4.8 | 0.56 | 11.7 |
| A66 | 4.87 | 0.35 | 7.2 |
| A67 | 4.87 | 0.35 | 7.2 |
| A68 | 4.93 | 0.26 | 5.2 |
| A69 | 4.93 | 0.26 | 5.2 |
| A70 | 4.2 | 0.77 | 18.4 |
| A71 | 4.73 | 0.7 | 14.8 |
| A72 | 4.67 | 0.49 | 10.4 |
| A73 | 4.53 | 0.74 | 16.4 |
| A74 | 4.73 | 0.46 | 9.7 |
| A75 | 4.93 | 0.26 | 5.2 |
| A76 | 4.8 | 0.41 | 8.6 |
| A77 | 5 | 0 | 0 |
| A78 | 4.67 | 0.72 | 15.4 |
| A79 | 4.67 | 0.72 | 15.4 |
| A80 | 4.87 | 0.35 | 7.2 |
| A81 | 3.73 | 1.39 | 37.2 |
| A82 | 3.47 | 1.3 | 37.5 |
| A83 | 3.47 | 1.06 | 30.5 |
| A84 | 4.93 | 0.26 | 5.2 |
| A85 | 4.93 | 0.26 | 5.2 |
| A86 | 4.8 | 0.41 | 8.6 |
| A87 | 3.93 | 1.16 | 29.5 |
| A88 | 4.13 | 0.99 | 24 |
| A89 | 4.33 | 0.9 | 20.8 |
| A90 | 4.07 | 1.22 | 30 |
| A91 | 4.73 | 0.46 | 9.7 |
| A92 | 4.67 | 0.72 | 15.4 |
| A93 | 4.27 | 1.03 | 24.1 |
| A94 | 4.93 | 0.26 | 5.2 |
| A95 | 4.53 | 0.83 | 18.4 |
| A96 | 4.73 | 0.7 | 14.8 |
| A97 | 4.87 | 0.35 | 7.2 |
| A98 | 4.67 | 0.72 | 15.4 |
| A99 | 4.27 | 1.22 | 28.6 |
| A100 | 4.93 | 0.26 | 5.2 |
| A101 | 4.73 | 0.59 | 12.5 |
| A102 | 4.73 | 0.7 | 14.8 |
